# Ohnut Versus a Waitlist Control for the Self-management of Endometriosis-Associated Deep Dyspareunia: Protocol for a Pilot Randomized Controlled Trial

**DOI:** 10.2196/39834

**Published:** 2023-03-27

**Authors:** Sandy X J Zhang, Rebecca G K MacLeod, Gurkiran Parmar, Natasha L Orr, Kate J Wahl, Heather Noga, Arianne Albert, Ryan Flannigan, Lori A Brotto, Paul J Yong

**Affiliations:** 1 Department of Obstetrics & Gynaecology University of British Columbia Vancouver, BC Canada; 2 Women's Health Research Institute British Columbia Women’s Hospital & Health Centre Vancouver, BC Canada; 3 Department of Urology University of British Columbia Vancouver, BC Canada; 4 British Columbia Women’s Centre for Pelvic Pain & Endometriosis British Columbia Women’s Hospital & Health Centre Vancouver, BC Canada

**Keywords:** endometriosis, dyspareunia, randomized controlled trial, self-management, pilot project, pilot study, women's health, pain, gynecology, gynecologist, sexual health, sexual medicine

## Abstract

**Background:**

Endometriosis-associated deep dyspareunia is associated with reduced sexual quality of life, lower self-esteem, and impaired sexual function.

**Objective:**

The primary objective is to assess the acceptability of a phallus length reducer (brand name: Ohnut [OhnutCo]), which is a buffer worn over the penis or a penetrating object to reduce endometriosis-associated deep dyspareunia, and the feasibility of a definitive randomized controlled trial (RCT). The secondary objective is to obtain estimates of the effectiveness of the buffer. An embedded substudy will explore the acceptability and the preliminary validity and reliability of a vaginal insert for the self-assessment of deep dyspareunia.

**Methods:**

Ours is an investigator-initiated, 2-arm RCT. We will recruit 40 patient participants with diagnosed endometriosis between the ages of 19 and 49 years, as well as their sexual partners. The participating couples will be randomized in a 1:1 ratio into the experimental arm or the waitlist control arm. The length of the study period will be 10 weeks, during which time all participants will record deep dyspareunia severity following each episode of sexual intercourse. In weeks 1 to 4, all patient participants will record deep dyspareunia severity at each sexual encounter. In weeks 5 to 10, participants in the experimental arm will use the buffer during vaginal penetration; participants in the waitlist control arm will continue engaging in vaginal penetration as usual. Participants will complete questionnaires for assessing measures of anxiety, depression, and sexual function at baseline, at 4 weeks, and at 10 weeks. In the substudy, patient participants will self-assess dyspareunia by using a vaginal insert on 2 occasions, at least 1 week apart. The primary outcomes—the acceptability and feasibility of the buffer—will be assessed with descriptive statistics, and the secondary outcome—phallus length reducer effectiveness—will be assessed by using an analysis of covariance–based approach. For the vaginal insert, we will assess acceptability, test-retest reliability, and convergent validity via correlation analyses comparing the use of the insert to clinical examination in terms of dyspareunia assessment outcomes.

**Results:**

Our pilot will provide initial data on the acceptability and effectiveness of the buffer and the feasibility of the study methodology. The results from our study are expected to be submitted for publication by the spring of 2023. As of September 2021, we have consented 31 couples into the study.

**Conclusions:**

Our study will provide preliminary evidence for the self-assessment and management of endometriosis-associated deep dyspareunia. The findings will inform the decision to proceed to a definitive RCT.

**Trial Registration:**

ClinicalTrials.gov NCT04370444; https://clinicaltrials.gov/ct2/show/NCT04370444

**International Registered Report Identifier (IRRID):**

DERR1-10.2196/39834

## Introduction

Endometriosis is the presence of endometrial-like glands and stromata that abnormally grow outside the uterus [1]. This disease affects approximately 1 in 10 women and can cause various types of pain, including dysmenorrhea; chronic pelvic pain; and deep dyspareunia, which is defined as pain with deep vaginal penetration [1,2]. Deep dyspareunia is often neglected due to its intimate nature and reservations among both patients and clinicians in discussing sexual health [3,4]. People with endometriosis and deep dyspareunia have been shown to have significantly reduced sexual quality of life [5], lower self-esteem [6], and impaired sexual function [7]. Qualitative research has shown that many people with deep dyspareunia feel guilty about their pain and often continue to engage in penetrative activity even when the pain is severe [6].

Endometriosis-associated deep dyspareunia is multifactorial and can result from the endometriotic lesions themselves and other factors, such as myofascial pelvic pain and central nervous system sensitization [1,2,8]. A subset of patients do not experience improvements in deep dyspareunia after standard care, such as hormonal or surgical interventions for endometriosis [7-11], indicating an important need for self-management approaches. One self-management option is a phallus length reducer (PLR; brand name: Ohnut [OhnutCo])—a buffer that is worn externally over the penis or a penetrating object to reduce the depth of penetration [9]. Conceptually, this should reduce deep dyspareunia by limiting contact with deep pelvic structures; however, this intervention has not been empirically tested.

The assessment of deep dyspareunia for clinical and research purposes is challenging; pelvic examination in the clinical context may not reliably reflect sexual activities, particularly for patients who have experienced trauma [12], while questionnaire-based measurement is limited by recall bias that may be exacerbated for patients who have become abstinent as a result of dyspareunia. A study that investigated superficial dyspareunia among people with vulvodynia found that the Tampon Test—a self-administered test involving the insertion and removal of a tampon—was reliable and demonstrated good construct validity [13]. The study showed 95% adherence, providing evidence that participants found the test to be an acceptable method of assessing superficial dyspareunia [13]. Similarly, the self-assessment of endometriosis-associated deep dyspareunia by using a vaginal insert may be an acceptable, ecologically valid approach to assessing this symptom.

In our pilot randomized controlled trial (RCT) protocol, the primary objective is to assess the acceptability of a PLR and the feasibility of a definitive RCT. The secondary objective is to obtain estimates of the effectiveness of the PLR, which will be compared to those of a waitlist control for reducing endometriosis-associated deep dyspareunia. An embedded substudy will explore the acceptability, reliability, and preliminary validity if a vaginal insert for the self-assessment of deep dyspareunia.

## Methods

### Trial Design

Our study is a pilot study of a parallel-group, 1:1 RCT of the PLR, which will be compared to a waitlist control. An embedded substudy will investigate the use of a vaginal insert (brand name: Soul Source Rigid Plastic Vaginal Dilator Size #P1 [Soul Source Therapeutic Devices Inc]) [14] for the self-assessment of deep dyspareunia.

This protocol was developed by using the SPIRIT (Standard Protocol Items: Recommendations for Interventional Trials) checklist [15], the CONSORT (Consolidated Standards of Reporting Trials) extension for randomized pilot and feasibility trials [16], and published guidance for the reporting of protocols of pilot and feasibility trials [17]. This protocol describes the study rationale, proposed methods, organization, and ethical considerations.

### Study Setting

The study is being conducted at a tertiary care center for endometriosis within a large urban center in western Canada. Our research team manages a prospective patient registry called *Endometriosis and Pelvic Pain Interdisciplinary Cohort* (EPPIC; ClinicalTrials.gov trial number: NCT02911090) [18]. Baseline data collected in EPPIC are derived from patient-reported questionnaires and clinician-reported data from clinic consultations. The variables that are included in this prospective registry have been described previously [18]. New or rereferral patients at the center are invited to participate in the registry and can consent to being contacted about further research. In our study, all procedures will be conducted by the participants at home.

### Eligibility Criteria

Eligible patient participants will be new or rereferred patients in EPPIC who attended a clinic visit between January 1, 2018, and December 31, 2020; those who consented to being contacted for future research; and those who have a history of surgically confirmed endometriosis. Details of the inclusion and exclusion criteria are listed in Textbox 1.

**Inclusion and exclusion criteria for the patient participants and their partners.**

**Patient participants**

- Inclusion criteria
  19 to 49 years of age
  Birth-assigned female
  Monogamous sexual relationship
  Sexually active or not currently sexually active due to deep dyspareunia
  Self-reported deep dyspareunia score of ≥4 (out of 10)
  Sexual partner who consents to participating in the study
  Willing to engage in penetrative sex at least once during the duration of the study
- Exclusion criteria
  Self-reported superficial dyspareunia score of ≥4 (out of 10; this is a potentially confounding variable, as the phallus length reducer is not expected to affect introital pain)
  Severe anxiety or depression symptoms in the last 2 weeks (Generalized Anxiety Disorder-7 or Patient Health Questionnaire-9 score of ≥15)
  Confirmed “yes” to question “In the last 2 weeks, have you had intense fear/anxiety in anticipation of, during or as a result of vaginal intercourse?”
  Current use of a phallus length reducer
  Inability to complete English-language questionnaires

**Partners**

- Inclusion criteria
  19 years of age or older
  Any sex and gender
- Exclusion criteria
  Current use of a phallus length reducer
  Inability to complete English-language questionnaires

### Sample Size

A sample size for a pilot study is often recommended to be at least 12 per group [19]. Given potential dropout and the uncertainty around the variance in acceptability, we will aim to recruit 20 participants and their sexual partners per group (ie, 40 couples or 80 total study participants).

### Recruitment

Individuals will be selected from a list of potential participants and will be telephoned and emailed about the study by a study coordinator. Interested individuals will be asked a series of screening questions to confirm that they meet the eligibility criteria. An important inclusion criterion listed in Textbox 1 is that participants must have a self-reported deep dyspareunia score of ≥4 (out of 10). This criterion was established to exclude patients who may have undergone surgery or other interventions that reduced deep dyspareunia symptoms. If the inclusion criteria are met and no exclusions are identified, patient participants and their partners will be emailed the consent form to review. The study coordinator will then review the consent forms with both the patient participants and their partners in a second telephone call and address any questions that the patient participants or their partners may have. Participants will give their consent to participate electronically, using an e-consent form.

### Interventions

The PLR (brand name: Ohnut) is made of a Food and Drug Administration–approved body- and skin-safe polymer blend. The blend is a thermo-set material that does not contain bisphenol A, phthalates, and latex and can be used with either silicone lubrication or water-based lubrication [20]. The PLR is a buffer that is worn externally over the penis or a penetrating object to limit the depth of penetration.

The vaginal insert is made of polyurethane and is stable from −40 to 70 °C. It will be self-inserted by the patient participants to self-assess the tenderness of pelvic structures implicated in deep dyspareunia that are typically assessed by a clinician during a pelvic examination. This is a novel, off-label use of the device.

To support the use of the vaginal insert and PLR, the study team developed patient-centered study booklets, which include instructions for the use of each tool (Multimedia Appendix 1). The instructions were developed with a gynecologist specialized in the assessment of dyspareunia, a pelvic floor physiotherapist with expertise in guiding patients to use vaginal inserts, and a clinical psychologist with experience in coaching patients and their partners to integrate the PLR in penetration. A medical illustrator was engaged to create diagrams for guiding the use of the vaginal insert.

### Outcomes

#### Primary Outcomes

The primary outcomes are the acceptability of the PLR and the feasibility of study methods. Details of the specific outcomes are listed in Textbox 2.

**Primary outcomes of the feasibility and acceptability of the phallus length reducer (PLR).**

**Outcomes and explanations**

- Response: the number of individuals who responded divided by the total number of individuals contacted
- Refusal: the number of individuals who refuse to participate divided by the number of individuals who responded
- Screen fail: the number of individuals who did not meet the inclusion criteria divided by the number of individuals who responded
- Recruitment rate: the number of participants enrolled divided by the length of the recruitment period (months)
- Retention: the number of participants who complete the study divided by the number of participants enrolled
- Adherence: the number of sexual events completed with the PLR divided by the total number of sexual events reported
- PLR acceptability: a 34-item, built-for-purpose questionnaire (adapted from a previously published measure for assessing the acceptability of female condoms [21]), with a response scale ranging from 1 to 5, will be completed by participants in the experimental arm in week 10 (Multimedia Appendix 1)

#### Secondary Outcomes

The secondary outcome is the effectiveness of the PLR, which will be determined by comparing changes in outcomes reported from week 4 to week 10 by patient participants in the experimental arm to those reported by participants in the control arm. Details of these outcomes are listed in Textbox 3.

**Secondary outcomes of the effectiveness of the phallus length reducer.**

**Outcomes and explanations**

- Deep dyspareunia: an 11-point numeric rating scale, wherein 0 corresponds to no pain and 10 corresponds to the worst pain, will be used to measure deep dyspareunia intensity (using an item previously published by our team [18]); mean dyspareunia scores from weeks 1 to 4 will be compared with those from weeks 5 to 10 in the control and experimental arms
- Sexual function: total score on the Female Sexual Function Index, a 19-item self-report measure that captures information on sexual desire, arousal, lubrication, orgasm, satisfaction, and pain over the past 4 weeks [22]
- Sexual distress: total score on the Female Sexual Distress Scale-Revised, a 13-item self-report measure that captures sex-related personal distress over the past 4 weeks [23]
- Anxiety: total score on the Generalized Anxiety Disorder-7, a 7-item questionnaire validated for monitoring the severity of general anxiety [24]
- Depression: total score on the Patient Health Questionnaire-9, a 9-item questionnaire validated for monitoring the severity of depression [25]

#### Substudy Outcomes

The substudy will explore the acceptability and the preliminary convergent validity and test-retest reliability of a vaginal insert for the self-assessment of deep dyspareunia. The vaginal insert acceptability substudy outcome is based on an 18-item, built-for-purpose questionnaire adapted from a previously published measure for assessing the acceptability of female condoms [21] on a response scale ranging from 1 to 5. This questionnaire will be completed by all patient participants by week 4 (Multimedia Appendix 1).

Participants will use the vaginal insert to palpate various pelvic organs and structures (ie, to palpate the bladder anteriorly, the pelvic floor laterally, the pouch of Douglas posteriorly, and the cervix at the apex of the vagina) that have been previously associated with deep dyspareunia [26], and they will indicate whether they feel pain (“yes” or “no”) and the severity of the pain (11-point numeric rating scale wherein 0 corresponds to no pain and 10 corresponds to the worst pain). The number of tender sites for each participant will be calculated based on the self-reported number of tender anatomic sites that the participants identify when using the insert. Data on clinically assessed tenderness (“yes” or “no”) for each participant were previously collected through digital pain mapping, which was completed by a clinician. Data for clinically assessed tenderness are stored in EPPIC. These pain-related variables will be used to assess the convergent validity and test-retest reliability of the vaginal insert.

### Assignment of Interventions

The study coordinator will use a REDCap (Research Electronic Data Capture; Vanderbilt University) extension package called *randomization* to randomly assign consented participants in a 1:1 ratio to 1 of 2 study arms; the experimental PLR arm will receive the PLR, and the waitlist control arm will receive the PLR once the study has concluded. Both arms will receive the vaginal insert for the substudy.

### Participant Timeline

Throughout the study, all participants will continue concurrent endometriosis treatments and treatments for other conditions as usual. At the start of the study, both arms will complete baseline demographic, psychological, and sexual function questionnaires. In weeks 1 to 4 of the study, patient participants in both arms will engage in sexual activity as usual and be asked to record a daily diary of all sexual events and the severity of deep dyspareunia (score: range 0-10). Psychological and sexual function questionnaires will be repeated at the end of week 4. In week 5, participants in the experimental arm will receive and begin to use the PLR. Participants in the waitlist control arm will not receive the PLR and will continue to record deep dyspareunia severity at each sexual encounter. At the end of week 10, all patient participants will repeat the psychological and sexual function questionnaires, and participants in the experimental arm will indicate the acceptability of the PLR. At the conclusion of the study period, participants in the waitlist control arm will receive the PLR.

For the substudy, during weeks 1 to 4, all patient participants will self-assess deep dyspareunia by using the vaginal insert on weeks 2 and 3 of their menstrual cycle or on another two occasions (1 week apart) in the absence of regular bleeding. At the end of week 4, all patient participants will indicate the acceptability of the vaginal insert. The participant timeline is depicted in Table 1.

**Table 1:** Schedule of interventions and assessments.

Groups and activities					Weeks 1 to 4							Weeks 5 to 10		
					Start of study		Vaginal insert self-assessment		Midstudy questionnaire		Experimental period			End of study
**Group 1 (PLR^a^ group)**														
	**Patients**													
		Informed consent		✓										
		Demographics form		✓										
		Study diary		✓		✓		✓		✓			✓	
		Vaginal insert self-assessment (twice)				✓								
		Receive and use PLR								✓				
		**Questionnaires**												
			FSFI^b^	✓				✓					✓	
			FSDS^c^	✓				✓					✓	
			PHQ-9^d^	✓				✓					✓	
			GAD-7^e^	✓				✓					✓	
			Vaginal insert feedback form					✓						
			PLR feedback form										✓	
	**Partners**													
		Informed consent		✓										
		Demographics form		✓										
		PLR feedback form											✓	
**Group 2 (control group)**														
	**Patients**													
		Informed consent		✓										
		Demographics form		✓										
		Study diary		✓		✓		✓		✓			✓	
		Vaginal insert self-assessment (twice)				✓								
		Continue sex as usual								✓				
		Receive PLR											✓	
		**Questionnaires**												
			FSFI	✓				✓					✓	
			FSDS	✓				✓					✓	
			PHQ-9	✓				✓					✓	
			GAD-7	✓				✓					✓	
			Vaginal insert feedback form					✓						
	**Partners**													
		Informed consent		✓										
		Demographics form		✓										

^a^PLR: phallus length reducer.

^b^FSFI: Female Sexual Function Index.

^c^FSDS: Female Sexual Distress Scale.

^d^PHQ-9: Patient Health Questionnaire-9.

^e^GAD-7: Generalized Anxiety Disorder-7.

### Confidentiality

The list of potential participants is password protected and stored on a secure server at the study site, and it will only be available to select members of the research team. Upon enrollment to the study, each participant will be given a unique study ID number that does not correlate to any personally identifying information. All reports, data collection sheets, and administrative forms will be identified by this number to maintain confidentiality. This ID number can only be decrypted by a password-protected linking file, which is stored separately on the secure server at the study site; similarly, it will only be available to select members of the research team.

### Data Management

Study materials, such as the instruction booklet, the daily diaries, and the PLR, will be mailed to the participants, depending on their study arm allocation. The study coordinator will follow up with each participant to collect the daily diaries of the control and intervention group participants upon the completion of the study. We will not blind the research team to the study arm allocations. Once the participants have completed the study, a research team member will extract the data from the daily diaries and upload the data onto a Microsoft Excel (Microsoft Corporation) sheet. The data will be stored on a secure server at the study site, and they will only be available to select members of the research team. Details of the data management procedures can be found in the study’s research ethics board application, which can be provided upon request. We do not anticipate any adverse events with the use of the PLR, and as such, we will not need a data monitoring committee. If any adverse events occur, the participants can record them in the daily diary, and they will have the contact information of the study team to debrief such cases.

### Statistical Methods

Descriptive statistics for demographic data and outcome variables will be presented by using means and SDs for continuous variables that are normally distributed, medians and IQRs for continuous variables that are not normally distributed, and frequency tables for the categorical variables of each study arm and the whole sample. Baseline measures will be compared to assess balance between the two arms, using Fisher exact tests for categorical variables and Kruskal-Wallis tests for continuous variables. With regard to our analytic approach for missing data.

### Acceptability of the PLR and Vaginal Insert

For the PLR and vaginal insert, an overall acceptability index will be calculated by summing the acceptability questionnaire item responses and dividing the sum by the total possible score. The index will be characterized by using means and SDs, and individual items will be reported by using medians and IQRs. For the PLR, results that are stratified by participant type (patient or partner) will be reported.

### Effectiveness of Using a PLR to Reduce Deep Dyspareunia

For the experimental and waitlist control arms, the difference in deep dyspareunia scores from week 4 to week 10 between the two arms will be assessed by using an analysis of covariance (ANCOVA)–based approach [27]. We will compare mean scores from weeks 5 to 10 between the two arms, using preintervention (weeks 1-4) mean scores as covariates in the model. Similar ANCOVA modeling will be used for the Female Sexual Distress Scale-Revised, Female Sexual Function Index, Generalized Anxiety Disorder-7, and Patient Health Questionnaire-9 scores. If the assumptions of the ANCOVA model are not met by the data set, then we will consider conducting other appropriate statistical analyses, such as the ANOVA-based approach, 2-tailed *t* tests, linear regression modeling, and nonparametric approaches for the ANCOVA model.

### Validity and Reliability of the Vaginal Insert for the Self-assessment of Deep Dyspareunia

Tenderness at each pelvic anatomic site, as assessed via the vaginal insert test, as well as the highest score across all anatomic locations, will be correlated with the mean deep dyspareunia score, highest deep dyspareunia score, and lowest deep dyspareunia score. The self-assessed tenderness scores will be correlated with self-reported deep dyspareunia scores, using the Spearman rank correlation test. Participants without sexual events during weeks 1 to 4 will be compared to participants with ≥1 sexual event.

The results of assessments of tenderness (“yes” or “no”) from the most recently performed clinical pelvic examinations, if applicable (these data are collected in EPPIC), will be correlated with self-assessed tenderness, as measured with the vaginal insert (score: range 0-10), by using a 2-tailed *t* test or Mann-Whitney *U* test.

To evaluate test-retest reliability, self-assessed tenderness scores for each anatomic site from the first assessment will be correlated with self-assessed tenderness scores from the second assessment to determine if the two sets of vaginal insert data are reproducible over time. This will be done by using scores (0-10) to calculate the intraclass correlation coefficient, as well as by categorizing tenderness (“yes” or “no”) and using the McNemar test.

With regard to our analytic approach for missing data, if the maximum amount of missingness in any variable is <5%, we will exclude cases with missing data via pairwise exclusion. Otherwise, if the maximum amount of missingness is >5%, multiple imputation via chained equations will be used to impute the missing values. Results from imputed data sets will be pooled to provide final results.

### Ethics Approval

The research presented in this protocol was approved by the Children’s and Women’s Research Ethics Board of the University of British Columbia (approval number: H19-00294). The study is also registered on ClinicalTrials.gov (trial number: NCT04370444). Any subsequent modifications to the study protocol will be reviewed by the Research Ethics Board of the University of British Columbia for approval, submitted to ClinicalTrials.gov, and communicated to the study participants.

## Results

The results from our study are expected to be submitted for publication by the spring of 2023. As of September 2021, we have recruited 31 couples into the study.

## Discussion

Deep dyspareunia affects approximately 50% of people with endometriosis and has significant impacts on their sexual and mental health [5-7]. One gap in the care of endometriosis-associated deep dyspareunia is the lack of evidence-based self-management tools. A second gap is the lack of validated self-assessment tools that patients can use for objective measurements of deep dyspareunia that can supplement patient-reported outcomes of pain severity. In this protocol, we propose a pilot RCT for evaluating the feasibility and acceptability of, and obtaining estimates of effectiveness for, a PLR (brand name: Ohnut), which is a buffer that limits the depth of penetration and can be used as a self-management tool for deep dyspareunia. Embedded in our pilot RCT is a substudy that will evaluate a vaginal insert for objectively self-assessing anatomic sites associated with deep dyspareunia.

Potential feasibility issues pertain to the recruitment rate, retention, and adherence. Both the PLR and vaginal insert will be used in vaginal penetrative activities. The vaginal insert will be used on 2 occasions (in order to test reproducibility), and the PLR will be used during sexual encounters involving vaginal penetration over 6 weeks. Moreover, pain scores will be prospectively recorded for each sexual encounter during the 6 weeks. These intensive procedures and prospective data collection activities may reduce the recruitment rate, retention, and adherence. Acceptability issues may include impacts on the sexual function of the partners and how the placement of the PLR on the penis or a penetrating object affects the sexual response cycles of the participants. We also aim to estimate the potential effectiveness of the PLR in reducing deep dyspareunia by comparing it to a waitlist control.

The strengths of the protocol include having a randomized design, taking prospective pain measurements at each sexual encounter, and supplementing the trial by embedding the vaginal insert as a potential novel self-assessment tool. One limitation of the protocol is that it is not placebo controlled; however, it would be difficult to design a placebo for the PLR. Another limitation is that we will be comparing the vaginal insert to clinical examinations that are done at entry into the registry, which may be conducted several months before enrollment into the trial. In a future study, a pelvic examination can also be performed at recruitment. Furthermore, a limitation of the pilot trial is that we will not control for variables like age and BMI, which are potential confounders that may affect the use of the vaginal insert or PLR.

In summary, the PLR may be a noninvasive self-management tool for people with endometriosis-associated deep dyspareunia. The results of our pilot RCT will inform a future full-scale RCT of both the PLR and the vaginal insert test. The results from our study will be reported in peer-reviewed journals, in conference presentations, and to the study participants and the public through plain-language knowledge translation strategies. Ultimately, these innovations will contribute to the body of work on the rigorous investigation and evidence-based management of endometriosis-associated deep dyspareunia.

## Appendix

Multimedia Appendix 1 Instructions for the control group and intervention group, as well as the acceptability questionnaires for the phallus length reducer and vaginal insert.

## Abbreviations

ANCOVA: analysis of covariance
CONSORT: Consolidated Standards of Reporting Trials
EPPIC: Endometriosis and Pelvic Pain Interdisciplinary Cohort
PLR: phallus length reducer
RCT: randomized controlled trial
REDCap: Research Electronic Data Capture
SPIRIT: Standard Protocol Items: Recommendations for Interventional Trials

## Data Availability

We do not have institutional ethics approval to publicly share the data sets in our study.
