# Supplementary material for: Ohnut Versus a Waitlist Control for the Self-management of Endometriosis-Associated Deep Dyspareunia: Protocol for a Pilot Randomized Controlled Trial
Source: JMIR Res Protoc. 2023 Mar 27;12:e39834. doi: 10.2196/39834 (PMC10131731; doi:10.2196/39834)

**Appendix A: Instructions for Control Group**

**Pathways for patient- centred diagnosis and management of endometriosis-associated deep dyspareunia: a pilot randomized trial**

**(PLR Study)**

**Study ID:**

**Table of Contents**

[Introduction 4](#_bookmark3)

[Using the purple insert to measure your pain with](#_bookmark4) [penetration 6](#_bookmark4)

[What are my measurement dates? 10](#_bookmark5)

[Daily diary 18](#_bookmark0)

[Thank you 29](#_bookmark1)

[Extra Tracking Sheets 30](#_bookmark2)

# Introduction

Thank you for agreeing to participate in this research study. As you know, we are trying to understand new approaches for managing the pain with deep penetration that can happen when you have endometriosis. This pain is called deep dyspareunia.

To do this, we are asking you to:

- Measure your own pain with penetration using a tool that we will call the **purple insert.**
- Continue having sex as per your usual for the duration of the study and keep a record in this study diary.

Here is the plan:

- During the first 4 weeks, use the insert to measure your pain with penetration and record the results in this diary (pages 10-16).
  - *If you have a regular period (e.g. approximately once a month)*, measure your pain once in the week after your period starts (week 2 of your cycle) and again 7 days later (week 3 of your cycle)
  - *If you do not have regular period (e.g. few irregular periods or no periods due to hormone treatment)*, measure your pain two times with a one week break between them

Page **4** of **32**

- At around the 4-week point, you will receive a second email titled “PLR Study Questionnaires Part 2”. Please complete the questionnaires linked in this email.
- At around the 10-week point, you will receive a third email titled “PLR Study Questionnaires Part 3”. Please complete the last set of questionnaires linked in this email.
- After week 10, you will get the phallus length reducer (PLR) in the mail to use during sex with your partner if you want.


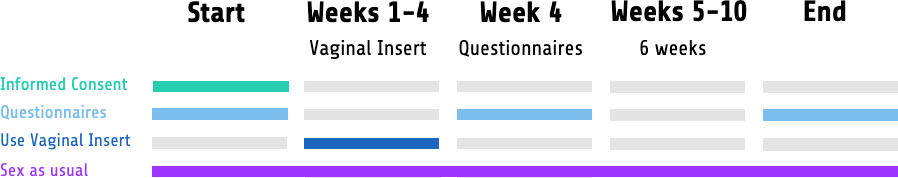


This diary includes all of the instructions and tracking forms you will need. If you have any questions for the research team, please email the study coordinator at [sandy.wu@cw.bc.ca](mailto:sandy.wu@cw.bc.ca).

This study is inclusive of all genders and sexual orientations. We use common terms like vagina, vulva, and penis in this diary and other study materials. We also respect the autonomy of participants in choosing other words used to describe their own bodies.

**Using the purple insert to measure your pain with penetration**

In this study, we want to understand whether people with endometriosis can measure their pain with deep penetration on their own. To do this, we are asking that you use the purple insert to press on areas that healthcare professionals usually check during pelvic exams.

***If you have a regular period*,** (e.g. approximately once a month) measure your pain once in the week after your period (week 2 of your cycle) and again 7 days later (week 3 of your cycle).

***If you do not have a regular period*,** (e.g. few irregular periods, or no periods due to hormonal treatment) measure your pain two times with a one week break between them.

**Before you start**

Set aside at least 20-30 minutes when you will not be interrupted. **Start by reading these instructions from start to finish.**

You will need the following items from your study box:

- This instruction sheet
- The purple insert
- Personal lubricant (do not use oil-based lube)
- Bumpy stickers

You will also need:

- A tissue or towel to wipe up any excess lubricant
- A mirror (optional, but might help you see)
- Pillows (to help support your back)
- A pen

**Setting up**

1. Take one sticker and place it near the wide end of the purple insert, in line with the white dots, as indicated by the white star in the picture below.


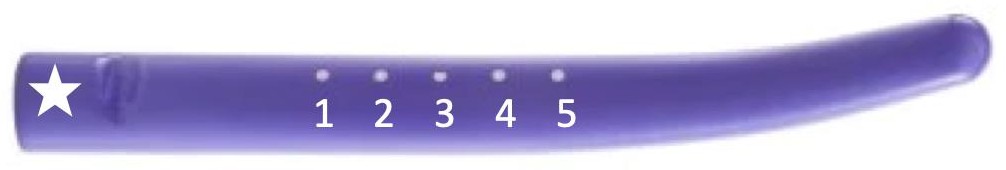


**Purple insert.** The insert has 5 white dots that you will use to measure how far inside the vagina it is. You will **not** be inserting the full length of the insert.

1. Prop yourself up semi-reclined on a bed/chair/sofa and bend your knees with your feet flat. For example, lean against a wall or headboard with your back propped up with some pillows. Try to avoid being in a crunch position (like a sit up) so you do not tense up.
2. If you like, you can place a small mirror in front of you, so you can see better, but try to avoid straining your neck or back trying to look in the mirror.
3. Relax your pelvic floor muscles by softening your stomach and widening your sit bones. This may feel similar to how your muscles relax when you let yourself pee and will make it easier to slide the insert into your vagina.
4. Generously apply lubricant onto the insert.

**Finding your home position**


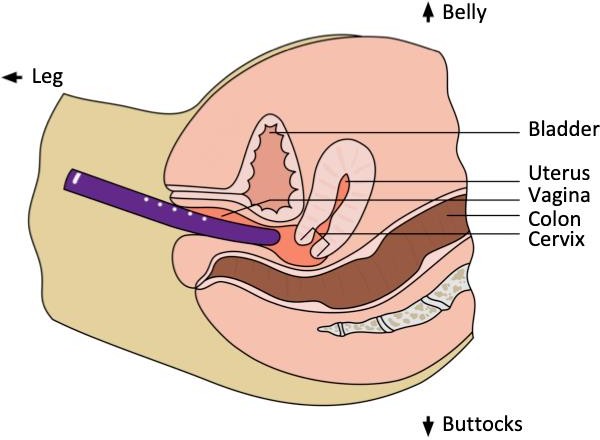


1. Gently slide the narrower, curved end of the purple insert into your vagina. If the insert slips out of your hand, just wipe off any excess lubricant and try again.
2. Slide the insert in as deep as you feel comfortable. For most people, this will be around dots 4-5 (with 1-3 dots remaining outside of your body).
3. Feel for the sticker you placed on the outside end of the insert.
4. Rotate the insert so the sticker is pointing up (towards the ceiling). This is your **home position**.
5. If at any time you feel that you are unsure which way the insert is pointing, just go back to home position.

Page **8** of **32**

**Measuring your pelvic pain**

Now that you are in the home position, you can move and angle the purple insert to gently press on 5 pelvic areas: your bladder, your right pelvic floor muscles, your left pelvic floor muscles, your cervix, and your cul-de- sac (the area at the top of the vagina between your cervix-uterus and bowel).

Try to press each area with the same amount of gentle pressure. For example, press the insert into the fleshy part of your palm under your thumb until you see a slight indent. This is about how much gentle pressure you should use when measuring your pelvic pain.

For each area, circle answer these questions on the following pages:

- - Does this area hurt to touch? (yes/no)
  - How much does this area hurt to touch from 0 (no pain) to 10 (worst pain imaginable)?
  - How many dots can you see on the insert (remaining outside your body)? (0-5)
- After touching each area with the purple insert, go back to the home position.
- If the insert slips out of your hand, go back to the home position, and try again.
- If at any point you feel unsafe, skip the area or stop doing the self-assessment.

Page **9** of **32**

**What are my measurement dates?**

If you have a regular period, please try to measure your pain during week 2 and week 3 of your cycle.

If you have an irregular period or no period, please try to measure your pain anytime in the 4 week period, with one week between each measurement (e.g., week 1 and week 2; or week 2 and week 3; or week 3 and week 4)

Please write the dates that you do the measurements here:

Date 1:

Date 2:

**Once you have completed measurements on both dates, please take a photo of pages 10 - 16 and email a copy to the research team.**

Page **10** of **32**


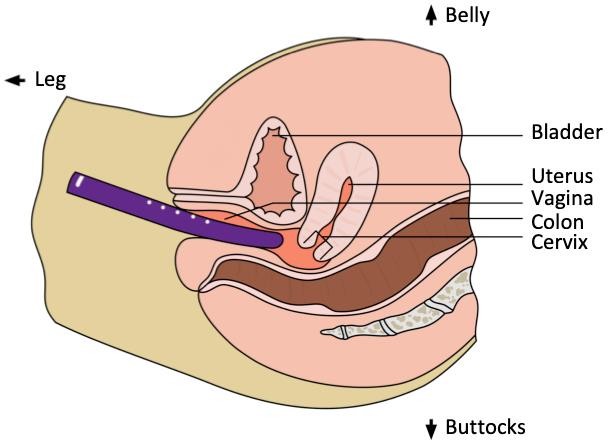
**Home position**

Gently slide the narrower, curved end of the insert into your vagina as deep as you feel comfortable.

Rotate the insert so the sticker is pointing up (12 o’clock).

|  | **Does this area hurt to touch?** | **How much does this area hurt to touch?** | | | | | | | | **How many dots can you see on the insert?** | | | | |
| --- | --- | --- | --- | --- | --- | --- | --- | --- | --- | --- | --- | --- | --- | --- |
| **Date 1** | **Y N** | **0 1**  (no pain) | **2** | **3** | **4** | **5** | **6** | **7** | **8 9 10**  (worst pain imaginable) | **1** | **2** | **3** | **4** | **5** |
| **Date 2** | **Y N** | **0 1** | **2** | **3** | **4** | **5** | **6** | **7** | **8 9 10** | **1** | **2** | **3** | **4** | **5** |


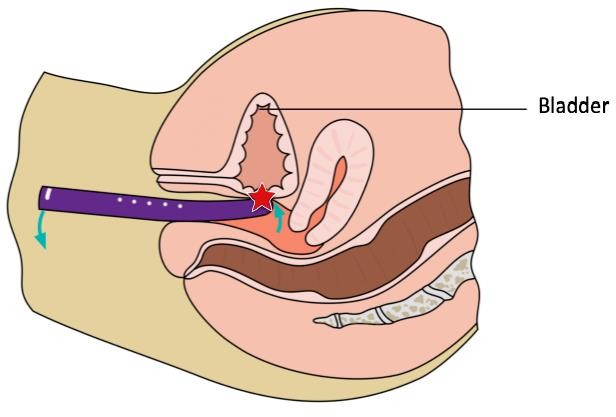
**Bladder**

From the home position, gently move the outside end of the insert that you are holding down towards your buttock.

This will cause the inside curve to press upward.

|  | **Does this area hurt to touch?** | **How much does this area hurt to touch?** | | | | | | | | **How many dots can you see on the insert?** | | | | |
| --- | --- | --- | --- | --- | --- | --- | --- | --- | --- | --- | --- | --- | --- | --- |
| **Date 1** | **Y N** | **0 1**  (no pain) | **2** | **3** | **4** | **5** | **6** | **7** | **8 9 10**  (worst pain imaginable) | **1** | **2** | **3** | **4** | **5** |
| **Date 2** | **Y N** | **0 1** | **2** | **3** | **4** | **5** | **6** | **7** | **8 9 10** | **1** | **2** | **3** | **4** | **5** |


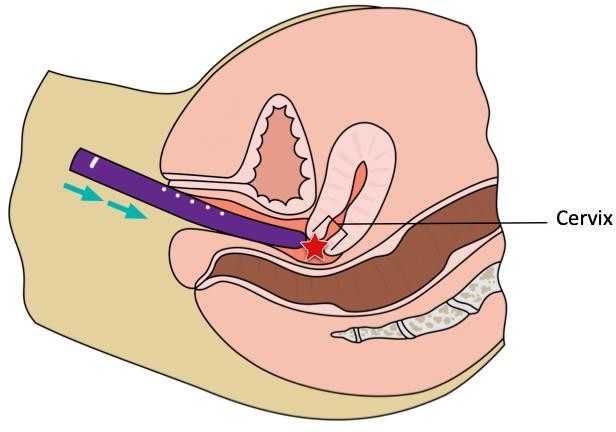
**Cervix-uterus**

From the home position, keep the insert completely straight so that the sticker is pointing up (12 o’clock).

Gently slide it straight in until you feel your cervix at the end of the vagina.

|  | **Does this area hurt to touch?** | **How much does this area hurt to touch?** | | | | | | | | **How many dots can you see on the insert?** | | | | |
| --- | --- | --- | --- | --- | --- | --- | --- | --- | --- | --- | --- | --- | --- | --- |
| **Date 1** | **Y N** | **0 1**  (no pain) | **2** | **3** | **4** | **5** | **6** | **7** | **8 9 10**  (worst pain imaginable) | **1** | **2** | **3** | **4** | **5** |
| **Date 2** | **Y N** | **0 1** | **2** | **3** | **4** | **5** | **6** | **7** | **8 9 10** | **1** | **2** | **3** | **4** | **5** |


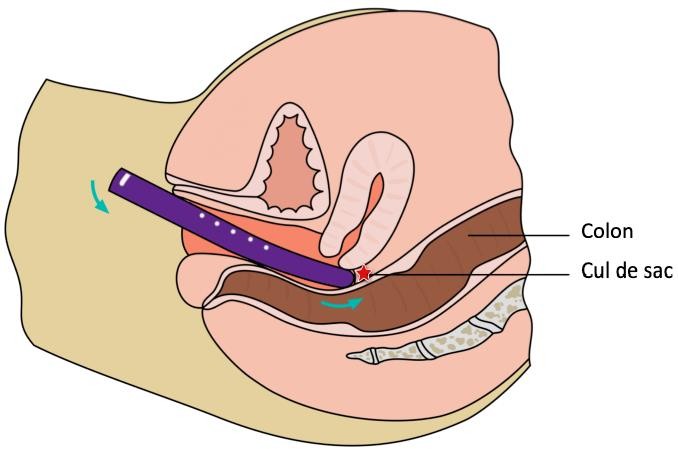
**Cul-de-sac**

From the home position, keep the insert completely straight so that the sticker is pointing up (12 o’clock).

Gently point the insert downwards, towards the back wall of the vagina. Then slide the insert straight in, along the back wall of the vagina, until you reach the cul-de-sac, which is under your cervix at the end of the vagina.

|  | **Does this area hurt to touch?** | **How much does this area hurt to touch?** | | | | | | | | **How many dots can you see on the insert?** | | | | |
| --- | --- | --- | --- | --- | --- | --- | --- | --- | --- | --- | --- | --- | --- | --- |
| **Date 1** | **Y N** | **0 1**  (no pain) | **2** | **3** | **4** | **5** | **6** | **7** | **8 9 10**  (worst pain imaginable) | **1** | **2** | **3** | **4** | **5** |
| **Date 2** | **Y N** | **0 1** | **2** | **3** | **4** | **5** | **6** | **7** | **8 9 10** | **1** | **2** | **3** | **4** | **5** |


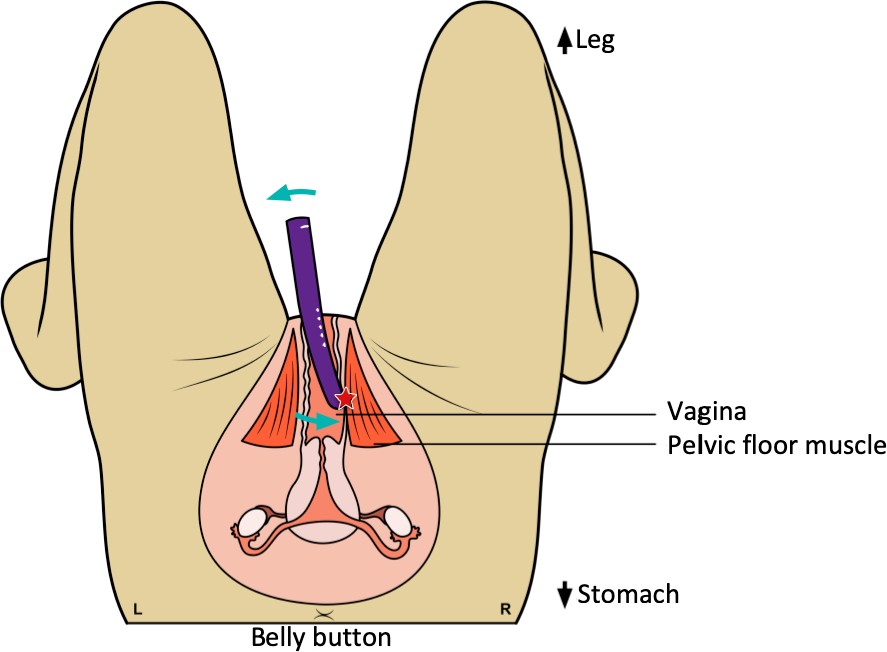
**Right pelvic floor**

From the home position, rotate the insert so that the sticker is pointing to your right (3 o’clock).

Gently move the outside end of the insert to your left. This will cause the inside curve to press towards your right pelvic floor muscles.

|  | **Does this area hurt to touch?** | **How much does this area hurt to touch?** | | | | | | | | **How many dots can you see on the insert?** | | | | |
| --- | --- | --- | --- | --- | --- | --- | --- | --- | --- | --- | --- | --- | --- | --- |
| **Date 1** | **Y N** | **0 1**  (no pain) | **2** | **3** | **4** | **5** | **6** | **7** | **8 9 10**  (worst pain imaginable) | **1** | **2** | **3** | **4** | **5** |
| **Date 2** | **Y N** | **0 1** | **2** | **3** | **4** | **5** | **6** | **7** | **8 9 10** | **1** | **2** | **3** | **4** | **5** |


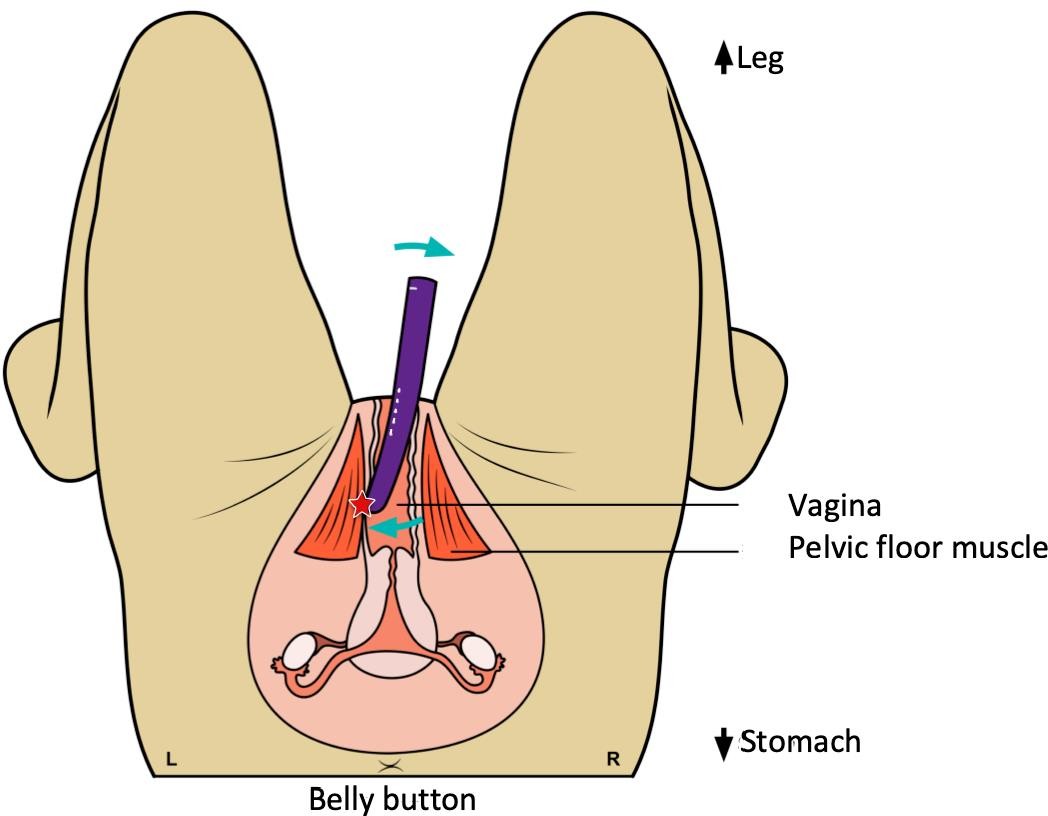
**Left pelvic floor**

From the home position, rotate the insert so that the sticker is pointing to your left (9 o’clock).

Gently move the outside end of the insert to your right. This will cause the inside curve to press towards your left pelvic floor muscles.

|  | **Does this area hurt to touch?** | **How much does this area hurt to touch?** | | | | | | | **How many dots can you see on the insert?** | | | | |
| --- | --- | --- | --- | --- | --- | --- | --- | --- | --- | --- | --- | --- | --- |
| **Date 1** | **Y N** | **0 1 2**  (no pain) | **3** | **4** | **5** | **6** | **7** | **8 9 10**  (worst pain imaginable) | **1** | **2** | **3** | **4** | **5** |
| **Date 2** | **Y N** | **0 1 2** | **3** | **4** | **5** | **6** | **7** | **8 9 10** | **1** | **2** | **3** | **4** | **5** |

**Thank you for completing the measurements! Once you have completed both sets of measurements, please take photos of pages 10 - 16 and email a copy to the research team.**

Page **17** of **32**

**Daily diary**

In this study, we want to understand whether using a PLR affects the pain with deep penetration that people with endometriosis experience. To do this, we are asking you to answer the questions in this diary every day for the next 10 weeks.

For these 10 weeks, you and your partner can have sex like you normally do. Please use the daily diary circle answer these questions on the following pages:

- Did you have sex? (yes/no)
- How painful was deep penetration? (0 - no pain, to 10

- worst pain imaginable, or N/A if you did not have sex that day)

- When we ask if you had sex, we are asking if you had specifically penetrative, vaginal sex (was something like a penis, fingers, or sex toy inserted inside your vagina).
- Fill out the diary every day, even if you and your partner do not have sex.
- On days when you and your partner do not have sex, only answer the question “Did you have sex?”
- If you make a mistake and need more pages, there are extras at the very end of this diary.

Page **18** of **32**

| **Week 2**  **DAY 1 DATE:** | | | | | | | | | | | | | |
| --- | --- | --- | --- | --- | --- | --- | --- | --- | --- | --- | --- | --- | --- |
|  | **Did you have sex?** | | **How painful was deep penetration?** | | | | | | | | | | |
| **Day 1** | **Yes** | **No** | **0**  (no pain) | **1** | **2** | **3** | **4** | **5** | **6** | **7** | **8** | **9** | **10**  (worst pain imaginable) |
| **Day 2** | **Yes** | **No** | **0** | **1** | **2** | **3** | **4** | **5** | **6** | **7** | **8** | **9** | **10** |
| **Day 3** | **Yes** | **No** | **0** | **1** | **2** | **3** | **4** | **5** | **6** | **7** | **8** | **9** | **10** |
| **Day 4** | **Yes** | **No** | **0** | **1** | **2** | **3** | **4** | **5** | **6** | **7** | **8** | **9** | **10** |
| **Day 5** | **Yes** | **No** | **0** | **1** | **2** | **3** | **4** | **5** | **6** | **7** | **8** | **9** | **10** |
| **Day 6** | **Yes** | **No** | **0** | **1** | **2** | **3** | **4** | **5** | **6** | **7** | **8** | **9** | **10** |
| **Day 7** | **Yes** | **No** | **0** | **1** | **2** | **3** | **4** | **5** | **6** | **7** | **8** | **9** | **10** |
| **Thank you for completing this week! Please take a photo of this page and email a copy to the research team.** | | | | | | | | | | | | | |

| **Week 4**  **DAY 1 DATE:** | | | | | | | | | | | | | |
| --- | --- | --- | --- | --- | --- | --- | --- | --- | --- | --- | --- | --- | --- |
|  | **Did you have sex?** | | **How painful was deep penetration?** | | | | | | | | | | |
| **Day 1** | **Yes** | **No** | **0**  (no pain) | **1** | **2** | **3** | **4** | **5** | **6** | **7** | **8** | **9** | **10**  (worst pain imaginable) |
| **Day 2** | **Yes** | **No** | **0** | **1** | **2** | **3** | **4** | **5** | **6** | **7** | **8** | **9** | **10** |
| **Day 3** | **Yes** | **No** | **0** | **1** | **2** | **3** | **4** | **5** | **6** | **7** | **8** | **9** | **10** |
| **Day 4** | **Yes** | **No** | **0** | **1** | **2** | **3** | **4** | **5** | **6** | **7** | **8** | **9** | **10** |
| **Day 5** | **Yes** | **No** | **0** | **1** | **2** | **3** | **4** | **5** | **6** | **7** | **8** | **9** | **10** |
| **Day 6** | **Yes** | **No** | **0** | **1** | **2** | **3** | **4** | **5** | **6** | **7** | **8** | **9** | **10** |
| **Day 7** | **Yes** | **No** | **0** | **1** | **2** | **3** | **4** | **5** | **6** | **7** | **8** | **9** | **10** |
| **Thank you for completing this week! Please take a photo of this page and email a copy to the research team.** | | | | | | | | | | | | | |

| **Week 1**  **DAY 1 DATE:** | | | | | | | | | | | | | |
| --- | --- | --- | --- | --- | --- | --- | --- | --- | --- | --- | --- | --- | --- |
|  | **Did you have sex?** | | **How painful was deep penetration?** | | | | | | | | | | |
| **Day 1** | **Yes** | **No** | **0**  (no pain) | **1** | **2** | **3** | **4** | **5** | **6** | **7** | **8** | **9** | **10**  (worst pain imaginable) |
| **Day 2** | **Yes** | **No** | **0** | **1** | **2** | **3** | **4** | **5** | **6** | **7** | **8** | **9** | **10** |
| **Day 3** | **Yes** | **No** | **0** | **1** | **2** | **3** | **4** | **5** | **6** | **7** | **8** | **9** | **10** |
| **Day 4** | **Yes** | **No** | **0** | **1** | **2** | **3** | **4** | **5** | **6** | **7** | **8** | **9** | **10** |
| **Day 5** | **Yes** | **No** | **0** | **1** | **2** | **3** | **4** | **5** | **6** | **7** | **8** | **9** | **10** |
| **Day 6** | **Yes** | **No** | **0** | **1** | **2** | **3** | **4** | **5** | **6** | **7** | **8** | **9** | **10** |
| **Day 7** | **Yes** | **No** | **0** | **1** | **2** | **3** | **4** | **5** | **6** | **7** | **8** | **9** | **10** |
| **Thank you for completing this week! Please take a photo of this page and email a copy to the research team.** | | | | | | | | | | | | | |

| **Week 3**  **DAY 1 DATE:** | | | | | | | | | | | | | |
| --- | --- | --- | --- | --- | --- | --- | --- | --- | --- | --- | --- | --- | --- |
|  | **Did you have sex?** | | **How painful was deep penetration?** | | | | | | | | | | |
| **Day 1** | **Yes** | **No** | **0**  (no pain) | **1** | **2** | **3** | **4** | **5** | **6** | **7** | **8** | **9** | **10**  (worst pain imaginable) |
| **Day 2** | **Yes** | **No** | **0** | **1** | **2** | **3** | **4** | **5** | **6** | **7** | **8** | **9** | **10** |
| **Day 3** | **Yes** | **No** | **0** | **1** | **2** | **3** | **4** | **5** | **6** | **7** | **8** | **9** | **10** |
| **Day 4** | **Yes** | **No** | **0** | **1** | **2** | **3** | **4** | **5** | **6** | **7** | **8** | **9** | **10** |
| **Day 5** | **Yes** | **No** | **0** | **1** | **2** | **3** | **4** | **5** | **6** | **7** | **8** | **9** | **10** |
| **Day 6** | **Yes** | **No** | **0** | **1** | **2** | **3** | **4** | **5** | **6** | **7** | **8** | **9** | **10** |
| **Day 7** | **Yes** | **No** | **0** | **1** | **2** | **3** | **4** | **5** | **6** | **7** | **8** | **9** | **10** |
| **Thank you for completing this week! Please take a photo of this page and email a copy to the research team.** | | | | | | | | | | | | | |

| **Week 5**  **DAY 1 DATE:** | | | | | | | | | | | | | |
| --- | --- | --- | --- | --- | --- | --- | --- | --- | --- | --- | --- | --- | --- |
|  | **Did you have sex?** | | **How painful was deep penetration?** | | | | | | | | | | |
| **Day 1** | **Yes** | **No** | **0**  (no pain) | **1** | **2** | **3** | **4** | **5** | **6** | **7** | **8** | **9** | **10**  (worst pain imaginable) |
| **Day 2** | **Yes** | **No** | **0** | **1** | **2** | **3** | **4** | **5** | **6** | **7** | **8** | **9** | **10** |
| **Day 3** | **Yes** | **No** | **0** | **1** | **2** | **3** | **4** | **5** | **6** | **7** | **8** | **9** | **10** |
| **Day 4** | **Yes** | **No** | **0** | **1** | **2** | **3** | **4** | **5** | **6** | **7** | **8** | **9** | **10** |
| **Day 5** | **Yes** | **No** | **0** | **1** | **2** | **3** | **4** | **5** | **6** | **7** | **8** | **9** | **10** |
| **Day 6** | **Yes** | **No** | **0** | **1** | **2** | **3** | **4** | **5** | **6** | **7** | **8** | **9** | **10** |
| **Day 7** | **Yes** | **No** | **0** | **1** | **2** | **3** | **4** | **5** | **6** | **7** | **8** | **9** | **10** |
| **Thank you for completing this week! Please take a photo of this page and email a copy to the research team.** | | | | | | | | | | | | | |

| **Week 6**  **DAY 1 DATE:** | | | | | | | | | | | | | |
| --- | --- | --- | --- | --- | --- | --- | --- | --- | --- | --- | --- | --- | --- |
|  | **Did you have sex?** | | **How painful was deep penetration?** | | | | | | | | | | |
| **Day 1** | **Yes** | **No** | **0**  (no pain) | **1** | **2** | **3** | **4** | **5** | **6** | **7** | **8** | **9** | **10**  (worst pain imaginable) |
| **Day 2** | **Yes** | **No** | **0** | **1** | **2** | **3** | **4** | **5** | **6** | **7** | **8** | **9** | **10** |
| **Day 3** | **Yes** | **No** | **0** | **1** | **2** | **3** | **4** | **5** | **6** | **7** | **8** | **9** | **10** |
| **Day 4** | **Yes** | **No** | **0** | **1** | **2** | **3** | **4** | **5** | **6** | **7** | **8** | **9** | **10** |
| **Day 5** | **Yes** | **No** | **0** | **1** | **2** | **3** | **4** | **5** | **6** | **7** | **8** | **9** | **10** |
| **Day 6** | **Yes** | **No** | **0** | **1** | **2** | **3** | **4** | **5** | **6** | **7** | **8** | **9** | **10** |
| **Day 7** | **Yes** | **No** | **0** | **1** | **2** | **3** | **4** | **5** | **6** | **7** | **8** | **9** | **10** |
| **Thank you for completing this week! Please take a photo of this page and email a copy to the research team.** | | | | | | | | | | | | | |

| **Week 7**  **DAY 1 DATE:** | | | | | | | | | | | | | |
| --- | --- | --- | --- | --- | --- | --- | --- | --- | --- | --- | --- | --- | --- |
|  | **Did you have sex?** | | **How painful was deep penetration?** | | | | | | | | | | |
| **Day 1** | **Yes** | **No** | **0**  (no pain) | **1** | **2** | **3** | **4** | **5** | **6** | **7** | **8** | **9** | **10**  (worst pain imaginable) |
| **Day 2** | **Yes** | **No** | **0** | **1** | **2** | **3** | **4** | **5** | **6** | **7** | **8** | **9** | **10** |
| **Day 3** | **Yes** | **No** | **0** | **1** | **2** | **3** | **4** | **5** | **6** | **7** | **8** | **9** | **10** |
| **Day 4** | **Yes** | **No** | **0** | **1** | **2** | **3** | **4** | **5** | **6** | **7** | **8** | **9** | **10** |
| **Day 5** | **Yes** | **No** | **0** | **1** | **2** | **3** | **4** | **5** | **6** | **7** | **8** | **9** | **10** |
| **Day 6** | **Yes** | **No** | **0** | **1** | **2** | **3** | **4** | **5** | **6** | **7** | **8** | **9** | **10** |
| **Day 7** | **Yes** | **No** | **0** | **1** | **2** | **3** | **4** | **5** | **6** | **7** | **8** | **9** | **10** |
| **Thank you for completing this week! Please take a photo of this page and email a copy to the research team.** | | | | | | | | | | | | | |

| **Week 8**  **DAY 1 DATE:** | | | | | | | | | | | | | |
| --- | --- | --- | --- | --- | --- | --- | --- | --- | --- | --- | --- | --- | --- |
|  | **Did you have sex?** | | **How painful was deep penetration?** | | | | | | | | | | |
| **Day 1** | **Yes** | **No** | **0**  (no pain) | **1** | **2** | **3** | **4** | **5** | **6** | **7** | **8** | **9** | **10**  (worst pain imaginable) |
| **Day 2** | **Yes** | **No** | **0** | **1** | **2** | **3** | **4** | **5** | **6** | **7** | **8** | **9** | **10** |
| **Day 3** | **Yes** | **No** | **0** | **1** | **2** | **3** | **4** | **5** | **6** | **7** | **8** | **9** | **10** |
| **Day 4** | **Yes** | **No** | **0** | **1** | **2** | **3** | **4** | **5** | **6** | **7** | **8** | **9** | **10** |
| **Day 5** | **Yes** | **No** | **0** | **1** | **2** | **3** | **4** | **5** | **6** | **7** | **8** | **9** | **10** |
| **Day 6** | **Yes** | **No** | **0** | **1** | **2** | **3** | **4** | **5** | **6** | **7** | **8** | **9** | **10** |
| **Day 7** | **Yes** | **No** | **0** | **1** | **2** | **3** | **4** | **5** | **6** | **7** | **8** | **9** | **10** |
| **Thank you for completing this week! Please take a photo of this page and email a copy to the research team.** | | | | | | | | | | | | | |

| **Week 9**  **DAY 1 DATE:** | | | | | | | | | | | | | |
| --- | --- | --- | --- | --- | --- | --- | --- | --- | --- | --- | --- | --- | --- |
|  | **Did you have sex?** | | **How painful was deep penetration?** | | | | | | | | | | |
| **Day 1** | **Yes** | **No** | **0**  (no pain) | **1** | **2** | **3** | **4** | **5** | **6** | **7** | **8** | **9** | **10**  (worst pain imaginable) |
| **Day 2** | **Yes** | **No** | **0** | **1** | **2** | **3** | **4** | **5** | **6** | **7** | **8** | **9** | **10** |
| **Day 3** | **Yes** | **No** | **0** | **1** | **2** | **3** | **4** | **5** | **6** | **7** | **8** | **9** | **10** |
| **Day 4** | **Yes** | **No** | **0** | **1** | **2** | **3** | **4** | **5** | **6** | **7** | **8** | **9** | **10** |
| **Day 5** | **Yes** | **No** | **0** | **1** | **2** | **3** | **4** | **5** | **6** | **7** | **8** | **9** | **10** |
| **Day 6** | **Yes** | **No** | **0** | **1** | **2** | **3** | **4** | **5** | **6** | **7** | **8** | **9** | **10** |
| **Day 7** | **Yes** | **No** | **0** | **1** | **2** | **3** | **4** | **5** | **6** | **7** | **8** | **9** | **10** |
| **Thank you for completing this week! Please take a photo of this page and email a copy to the research team.** | | | | | | | | | | | | | |

| **Week 10**  **DAY 1 DATE:** | | | | | | | | | | | | | |
| --- | --- | --- | --- | --- | --- | --- | --- | --- | --- | --- | --- | --- | --- |
|  | **Did you have sex?** | | **How painful was deep penetration?** | | | | | | | | | | |
| **Day 1** | **Yes** | **No** | **0**  (no pain) | **1** | **2** | **3** | **4** | **5** | **6** | **7** | **8** | **9** | **10**  (worst pain imaginable) |
| **Day 2** | **Yes** | **No** | **0** | **1** | **2** | **3** | **4** | **5** | **6** | **7** | **8** | **9** | **10** |
| **Day 3** | **Yes** | **No** | **0** | **1** | **2** | **3** | **4** | **5** | **6** | **7** | **8** | **9** | **10** |
| **Day 4** | **Yes** | **No** | **0** | **1** | **2** | **3** | **4** | **5** | **6** | **7** | **8** | **9** | **10** |
| **Day 5** | **Yes** | **No** | **0** | **1** | **2** | **3** | **4** | **5** | **6** | **7** | **8** | **9** | **10** |
| **Day 6** | **Yes** | **No** | **0** | **1** | **2** | **3** | **4** | **5** | **6** | **7** | **8** | **9** | **10** |
| **Day 7** | **Yes** | **No** | **0** | **1** | **2** | **3** | **4** | **5** | **6** | **7** | **8** | **9** | **10** |
| **Thank you for completing this week! Please take a photo of this page and email a copy to the research team.** | | | | | | | | | | | | | |

**Thank you**

You have made it to the end of the study! We know that sharing information about your sex life and trying tools to manage pain with deep penetration can feel personal - we are very grateful that you agreed to do this research. We hope that the results of this project will provide knowledge that helps make dealing with pain easier for people with endometriosis in the future.

Before you finish up, please double check to make sure you have:

- Sent copies of all of your pain measurements (pages 10 - 16) and daily diary (pages 19 - 28) to the research team
- Finished all the questionnaires that the research team emailed you

Thank you from the whole research team!

Page **29** of **32**

**Extra Tracking Sheets**

| **Extra sheet**  **DAY 1 DATE:** | | | | | | | | | | | | | |
| --- | --- | --- | --- | --- | --- | --- | --- | --- | --- | --- | --- | --- | --- |
|  | **Did you have sex?** | | **How painful was deep penetration?** | | | | | | | | | | |
| **Day 1** | **Yes** | **No** | **0**  (no pain) | **1** | **2** | **3** | **4** | **5** | **6** | **7** | **8** | **9** | **10**  (worst pain imaginable) |
| **Day 2** | **Yes** | **No** | **0** | **1** | **2** | **3** | **4** | **5** | **6** | **7** | **8** | **9** | **10** |
| **Day 3** | **Yes** | **No** | **0** | **1** | **2** | **3** | **4** | **5** | **6** | **7** | **8** | **9** | **10** |
| **Day 4** | **Yes** | **No** | **0** | **1** | **2** | **3** | **4** | **5** | **6** | **7** | **8** | **9** | **10** |
| **Day 5** | **Yes** | **No** | **0** | **1** | **2** | **3** | **4** | **5** | **6** | **7** | **8** | **9** | **10** |
| **Day 6** | **Yes** | **No** | **0** | **1** | **2** | **3** | **4** | **5** | **6** | **7** | **8** | **9** | **10** |
| **Day 7** | **Yes** | **No** | **0** | **1** | **2** | **3** | **4** | **5** | **6** | **7** | **8** | **9** | **10** |
| **Thank you for completing this week! Please take a photo of this page and email a copy to the research team.** | | | | | | | | | | | | | |

| **Extra sheet**  **DAY 1 DATE:** | | | | | | | | | | | | | |
| --- | --- | --- | --- | --- | --- | --- | --- | --- | --- | --- | --- | --- | --- |
|  | **Did you have sex?** | | **How painful was deep penetration?** | | | | | | | | | | |
| **Day 1** | **Yes** | **No** | **0**  (no pain) | **1** | **2** | **3** | **4** | **5** | **6** | **7** | **8** | **9** | **10**  (worst pain imaginable) |
| **Day 2** | **Yes** | **No** | **0** | **1** | **2** | **3** | **4** | **5** | **6** | **7** | **8** | **9** | **10** |
| **Day 3** | **Yes** | **No** | **0** | **1** | **2** | **3** | **4** | **5** | **6** | **7** | **8** | **9** | **10** |
| **Day 4** | **Yes** | **No** | **0** | **1** | **2** | **3** | **4** | **5** | **6** | **7** | **8** | **9** | **10** |
| **Day 5** | **Yes** | **No** | **0** | **1** | **2** | **3** | **4** | **5** | **6** | **7** | **8** | **9** | **10** |
| **Day 6** | **Yes** | **No** | **0** | **1** | **2** | **3** | **4** | **5** | **6** | **7** | **8** | **9** | **10** |
| **Day 7** | **Yes** | **No** | **0** | **1** | **2** | **3** | **4** | **5** | **6** | **7** | **8** | **9** | **10** |
| **Thank you for completing this week! Please take a photo of this page and email a copy to the research team.** | | | | | | | | | | | | | |

| **Extra sheet**  **DAY 1 DATE:** | | | | | | | | | | | | | |
| --- | --- | --- | --- | --- | --- | --- | --- | --- | --- | --- | --- | --- | --- |
|  | **Did you have sex?** | | **How painful was deep penetration?** | | | | | | | | | | |
| **Day 1** | **Yes** | **No** | **0**  (no pain) | **1** | **2** | **3** | **4** | **5** | **6** | **7** | **8** | **9** | **10**  (worst pain imaginable) |
| **Day 2** | **Yes** | **No** | **0** | **1** | **2** | **3** | **4** | **5** | **6** | **7** | **8** | **9** | **10** |
| **Day 3** | **Yes** | **No** | **0** | **1** | **2** | **3** | **4** | **5** | **6** | **7** | **8** | **9** | **10** |
| **Day 4** | **Yes** | **No** | **0** | **1** | **2** | **3** | **4** | **5** | **6** | **7** | **8** | **9** | **10** |
| **Day 5** | **Yes** | **No** | **0** | **1** | **2** | **3** | **4** | **5** | **6** | **7** | **8** | **9** | **10** |
| **Day 6** | **Yes** | **No** | **0** | **1** | **2** | **3** | **4** | **5** | **6** | **7** | **8** | **9** | **10** |
| **Day 7** | **Yes** | **No** | **0** | **1** | **2** | **3** | **4** | **5** | **6** | **7** | **8** | **9** | **10** |
| **Thank you for completing this week! Please take a photo of this page and email a copy to the research team.** | | | | | | | | | | | | | |

**Appendix A: Instructions for Intervention Group**

**Pathways for patient- centred diagnosis and management of endometriosis-associated deep dyspareunia: a pilot randomized trial**

**(PLR Study)**

**Study ID:**

**Table of Contents**

[Introduction 4](#_TOC_250007)

[Using the purple insert to measure your pain with penetration 6](#_TOC_250006)

[What are my measurement dates? 10](#_TOC_250005)

[Daily Diary Instructions 17](#_TOC_250004)

[Daily Diary: Weeks 1-4 20](#_TOC_250003)

[PLR Instructions 24](#_TOC_250002)

[Daily Diary: Weeks 5-10 26](#_TOC_250001)

[Thank you 32](#_bookmark5)

[Extra Tracking Sheets 33](#_TOC_250000)

# Introduction

Thank you for agreeing to participate in this research study. As you know, we are trying to understand new approaches for managing the pain with deep penetration that can happen when you have endometriosis. This pain is called deep dyspareunia.

To do this, we are asking you to try two things:

- Measuring your own pain with penetration using a tool that we will call the **purple insert.**
- Using a **phallus length reducer (PLR)** during sex with your partner to see what effect it has on your pain with deep penetration.

Here is the plan:

- You should have already received an email titled “PLR Study Questionnaires Part 1”.
  - If you have not yet done so, please complete the questionnaires in this email.
- Have sex as you usually would for the first 4 weeks (i.e. week 1 to week 4) and keep track of any pain with deep penetration in this diary (pages 20 - 23).
- During those first 4 weeks, use the purple insert to measure your pain with penetration and record the results in this diary (pages 10 – 16).
  - *If you have a regular period (e.g. approximately once a month)*, measure your pain once in the week after your period starts (week 2 of your cycle) and again 7 days later (week 3 of your cycle)

o *If you do not have regular period (e.g. few irregular periods or no periods due to hormone treatment)*, measure your pain two times with a one week break between them

- At around the 4-week point, you will receive a second email titled “PLR Study Questionnaires Part 2”. Please complete the questionnaires in this email.
- During the next 6 weeks (week 5 to week 10), have sex using the PLR (it will arrive in the mail soon) and keep track of any pain with deep penetration in this diary (pages 26 – 31).
- At around the 10-week point, you will receive a third email titled “PLR Study Questionnaires Part 3”. Please complete the last set of questionnaires linked in this email.

This diary includes all of the instructions and tracking forms you will need. If you have any questions for the research team, please email the study coordinator at [sandy.wu@cw.bc.ca](mailto:sandy.wu@cw.bc.ca).


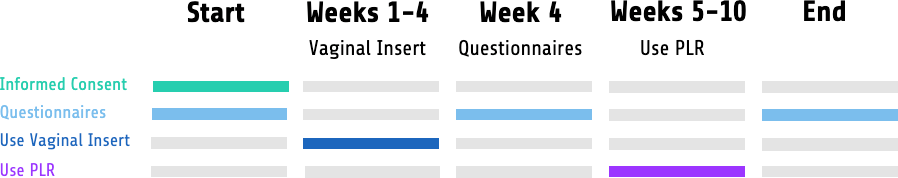


This study is inclusive of all genders and sexual orientations. We use common terms like vagina, vulva, and penis in this diary and other study materials. We also respect the autonomy of participants in choosing other words used to describe their own bodies.

**Using the purple insert to measure your pain with penetration**

In this study, we want to understand whether people with endometriosis can measure their pain with deep penetration on their own. To do this, we are asking that you use the purple insert to press on areas that healthcare professionals usually check during pelvic exams.

***If you have a regular period*,** (e.g. approximately once a month) measure your pain once in the week after your period (week 2 of your cycle) and again 7 days later (week 3 of your cycle).

***If you do not have a regular period*,** (e.g. few irregular periods, or no periods due to hormonal treatment) measure your pain two times with a one week break between them.

**Before you start**

Set aside at least 20-30 minutes when you will not be interrupted. **Start by reading these instructions from start to finish.**

You will need the following items from your study box:

- This instruction sheet
- The purple insert
- Personal lubricant (do not use oil-based lube)
- Bumpy stickers

Page **6** of **36**

You will also need:

- - A tissue or towel to wipe up any excess lubricant
  - A mirror (optional, but might help you see)
  - Pillows (to help support your back)
  - A pen

**Setting up**

1. Take one sticker and place it near the wide end of the purple insert, in line with the white dots, as indicated by the white star in the picture below.


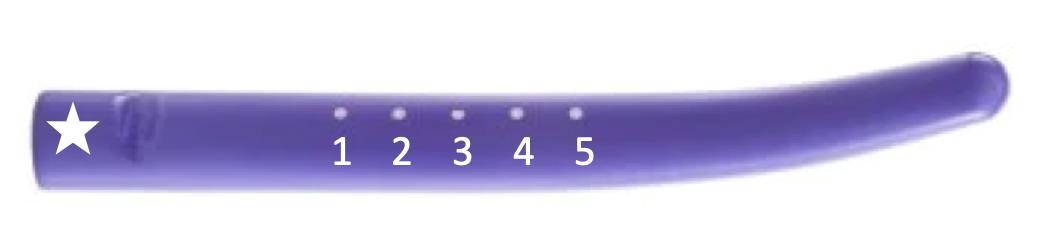


**Purple insert.** The insert has 5 white dots that you will use to measure how far inside the vagina it is. You will **not** be inserting the full length of the insert.

1. Prop yourself up semi-reclined on a bed/chair/sofa and bend your knees with your feet flat. For example, lean against a wall or headboard with your back propped up with some pillows. Try to avoid being in a crunch position (like a sit up) so you do not tense up.
2. If you like, you can place a small mirror in front of you, so you can see better, but try to avoid straining your neck or back trying to look in the mirror.
3. Relax your pelvic floor muscles by softening your stomach and widening your sit bones. This may feel similar to how your muscles relax when you let yourself pee and will make it easier to slide the insert into your vagina.
4. Generously apply lubricant onto the insert.

Page **7** of **36**

**Finding your home position**


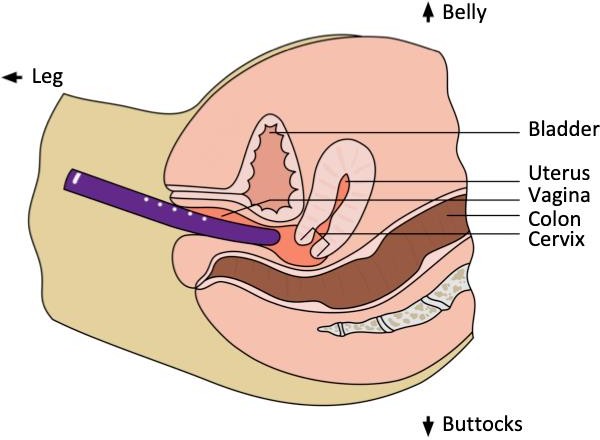


1. Gently slide the narrower, curved end of the purple insert into your vagina. If the insert slips out of your hand, just wipe off any excess lubricant and try again.
2. Slide the insert in as deep as you feel comfortable. For most people, this will be around dots 4-5 (with 1-3 dots remaining outside of your body).
3. Feel for the sticker you placed on the outside end of the insert.
4. Rotate the insert so the sticker is pointing up (towards the ceiling). This is your **home position**.
5. If at any time you feel that you are unsure which way the insert is pointing, just go back to home position.

Page **8** of **36**

**Measuring your pelvic pain**

Now that you are in the home position, you can move and angle the purple insert to gently press on 5 pelvic areas: your bladder, your right pelvic floor muscles, your left pelvic floor muscles, your cervix, and your cul-de- sac (the area at the top of the vagina between your cervix-uterus and bowel).

Try to press each area with the same amount of gentle pressure. For example, press the insert into the fleshy part of your palm under your thumb until you see a slight indent. This is about how much gentle pressure you should use when measuring your pelvic pain.

For each area, circle answer these questions on the following pages:

- - Does this area hurt to touch? (yes/no)
  - How much does this area hurt to touch from 0 (no pain) to 10 (worst pain imaginable)?
  - How many dots can you see on the insert (remaining outside your body)? (0-5)
- After touching each area with the purple insert, go back to the home position.
- If the insert slips out of your hand, go back to the home position, and try again.
- If at any point you feel unsafe, skip the area or stop doing the self-assessment.

Page **9** of **36**

**What are my measurement dates?**

If you have a regular period, please try to measure your pain during week 2 and week 3 of your cycle.

If you have an irregular period or no period, please try to measure your pain anytime in the 4 week period, with one week between each measurement (e.g., week 1 and week 2; or week 2 and week 3; or week 3 and week 4)

Please write the dates that you do the measurements here:

Date 1:

Date 2:

**Once you have completed measurements on both dates, please take a photo of page 10 - 16 and email a copy to the research team.**

Page **10** of **36**


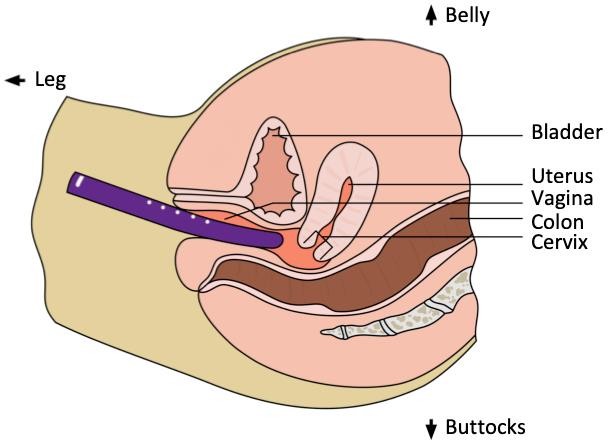
**Home position**

Gently slide the narrower, curved end of the insert into your vagina as deep as you feel comfortable.

Rotate the insert so the sticker is pointing up (12 o’clock).

|  | **Does this area hurt to touch?** | **How much does this area hurt to touch?** | | | | | | | | **How many dots can you see on the insert?** | | | | |
| --- | --- | --- | --- | --- | --- | --- | --- | --- | --- | --- | --- | --- | --- | --- |
| **Date 1** | **Y N** | **0 1**  (no pain) | **2** | **3** | **4** | **5** | **6** | **7** | **8 9 10**  (worst pain imaginable) | **1** | **2** | **3** | **4** | **5** |
| **Date 2** | **Y N** | **0 1** | **2** | **3** | **4** | **5** | **6** | **7** | **8 9 10** | **1** | **2** | **3** | **4** | **5** |


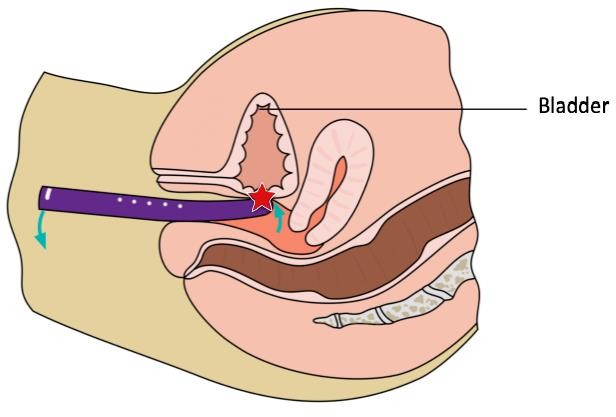
**Bladder**

From the home position, gently move the outside end of the insert that you are holding down towards your buttock.

This will cause the inside curve to press upward.

|  | **Does this area hurt to touch?** | **How much does this area hurt to touch?** | | | | | | | | **How many dots can you see on the insert?** | | | | |
| --- | --- | --- | --- | --- | --- | --- | --- | --- | --- | --- | --- | --- | --- | --- |
| **Date 1** | **Y N** | **0 1**  (no pain) | **2** | **3** | **4** | **5** | **6** | **7** | **8 9 10**  (worst pain imaginable) | **1** | **2** | **3** | **4** | **5** |
| **Date 2** | **Y N** | **0 1** | **2** | **3** | **4** | **5** | **6** | **7** | **8 9 10** | **1** | **2** | **3** | **4** | **5** |


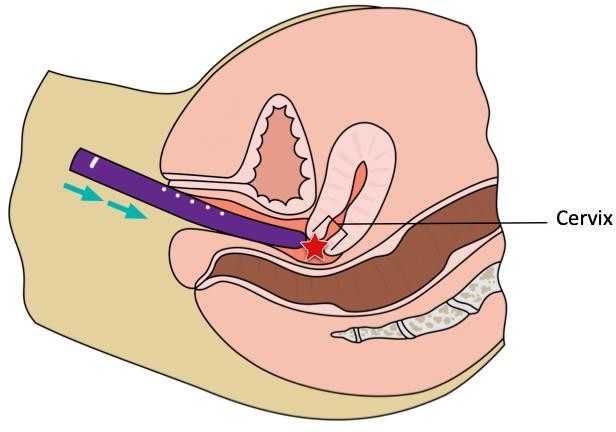
**Cervix-uterus**

From the home position, keep the insert completely straight so that the sticker is pointing up (12 o’clock).

Gently slide it straight in until you feel your cervix at the end of the vagina.

|  | **Does this area hurt to touch?** | **How much does this area hurt to touch?** | | | | | | | | **How many dots can you see on the insert?** | | | | |
| --- | --- | --- | --- | --- | --- | --- | --- | --- | --- | --- | --- | --- | --- | --- |
| **Date 1** | **Y N** | **0 1**  (no pain) | **2** | **3** | **4** | **5** | **6** | **7** | **8 9 10**  (worst pain imaginable) | **1** | **2** | **3** | **4** | **5** |
| **Date 2** | **Y N** | **0 1** | **2** | **3** | **4** | **5** | **6** | **7** | **8 9 10** | **1** | **2** | **3** | **4** | **5** |


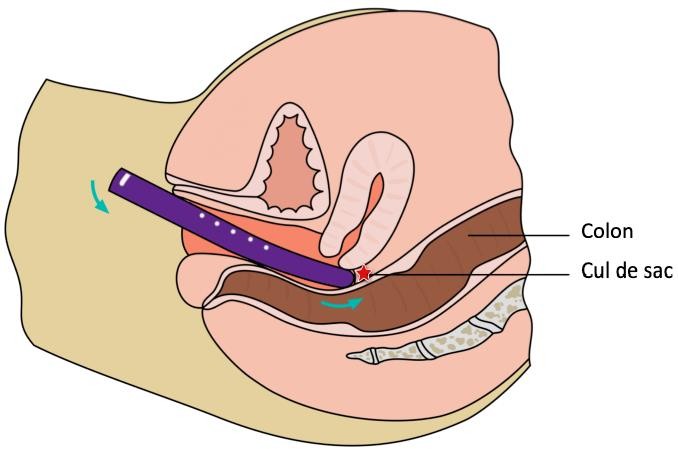
**Cul-de-sac**

From the home position, keep the insert completely straight so that the sticker is pointing up (12 o’clock).

Gently point the insert downwards, towards the back wall of the vagina. Then slide the insert straight in, along the back wall of the vagina, until you reach the cul-de-sac, which is under your cervix at the end of the vagina.

|  | **Does this area hurt to touch?** | **How much does this area hurt to touch?** | | | | | | | | **How many dots can you see on the insert?** | | | | |
| --- | --- | --- | --- | --- | --- | --- | --- | --- | --- | --- | --- | --- | --- | --- |
| **Date 1** | **Y N** | **0 1**  (no pain) | **2** | **3** | **4** | **5** | **6** | **7** | **8 9 10**  (worst pain imaginable) | **1** | **2** | **3** | **4** | **5** |
| **Date 2** | **Y N** | **0 1** | **2** | **3** | **4** | **5** | **6** | **7** | **8 9 10** | **1** | **2** | **3** | **4** | **5** |


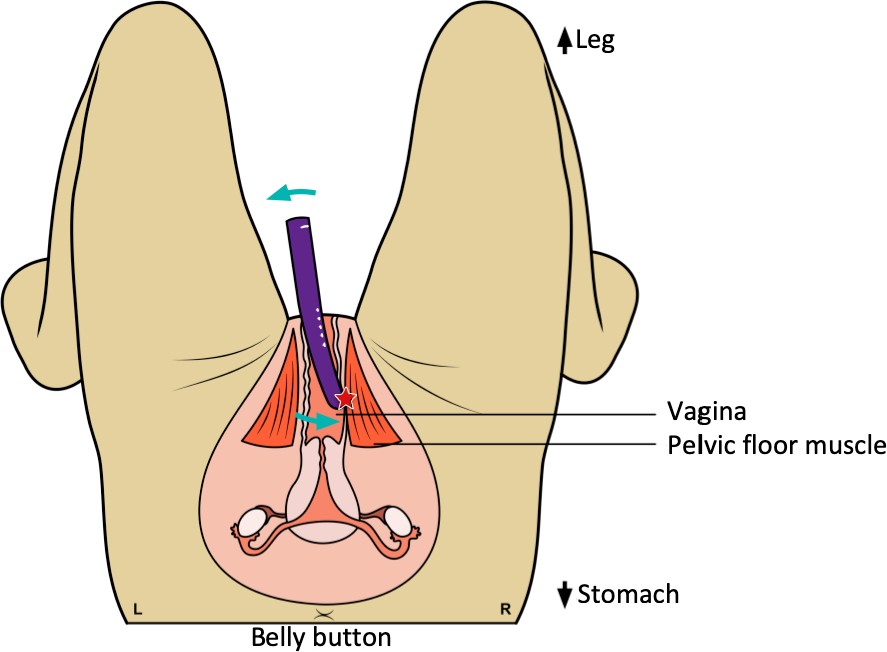
**Right pelvic floor**

From the home position, rotate the insert so that the sticker is pointing to your right (3 o’clock).

Gently move the outside end of the insert to your left. This will cause the inside curve to press towards your right pelvic floor muscles.

|  | **Does this area hurt to touch?** | **How much does this area hurt to touch?** | | | | | | | | **How many dots can you see on the insert?** | | | | |
| --- | --- | --- | --- | --- | --- | --- | --- | --- | --- | --- | --- | --- | --- | --- |
| **Date 1** | **Y N** | **0 1**  (no pain) | **2** | **3** | **4** | **5** | **6** | **7** | **8 9 10**  (worst pain imaginable) | **1** | **2** | **3** | **4** | **5** |
| **Date 2** | **Y N** | **0 1** | **2** | **3** | **4** | **5** | **6** | **7** | **8 9 10** | **1** | **2** | **3** | **4** | **5** |


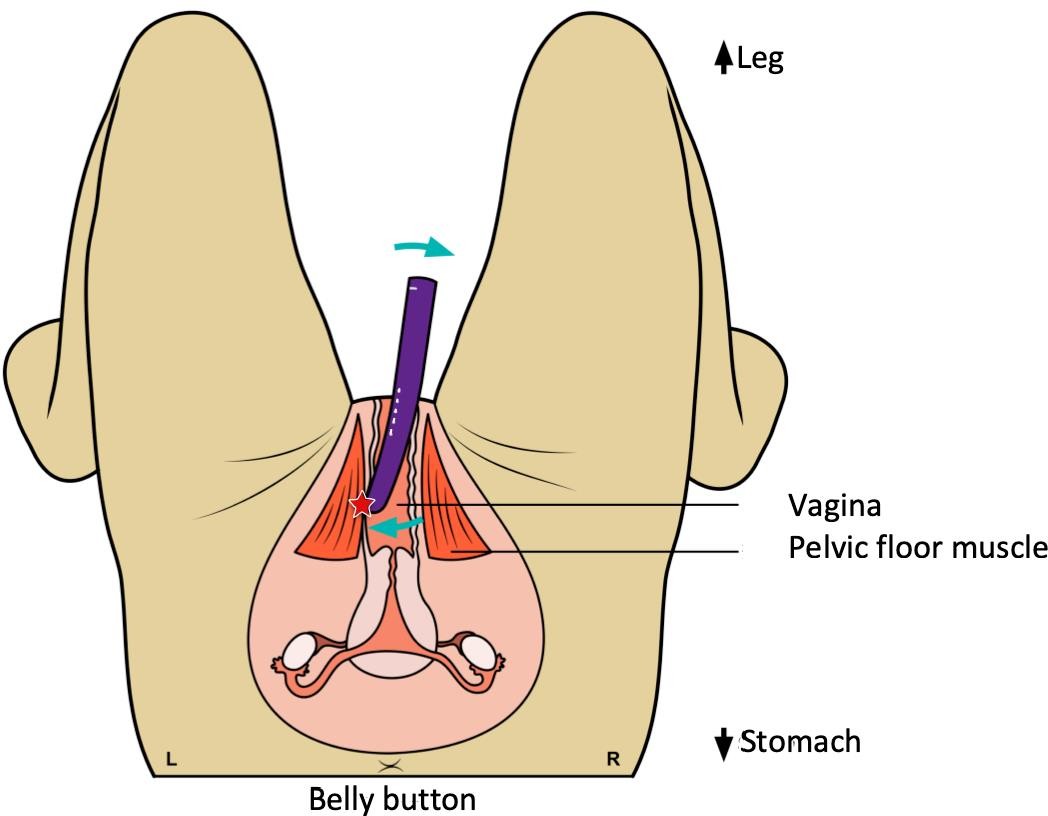
**Left pelvic floor**

From the home position, rotate the insert so that the sticker is pointing to your left (9 o’clock).

Gently move the outside end of the insert to your right. This will cause the inside curve to press towards your left pelvic floor muscles.

|  | **Does this area hurt to touch?** | **How much does this area hurt to touch?** | | | | | | | **How many dots can you see on the insert?** | | | | |
| --- | --- | --- | --- | --- | --- | --- | --- | --- | --- | --- | --- | --- | --- |
| **Date 1** | **Y N** | **0 1 2**  (no pain) | **3** | **4** | **5** | **6** | **7** | **8 9 10**  (worst pain imaginable) | **1** | **2** | **3** | **4** | **5** |
| **Date 2** | **Y N** | **0 1 2** | **3** | **4** | **5** | **6** | **7** | **8 9 10** | **1** | **2** | **3** | **4** | **5** |

**Thank you for completing the measurements! Once you have completed both sets of measurements, please take photos of pages 10 – 16, 20 - 23 and email a copy to the research team.**

Page **17** of **36**

**Daily Diary Instructions**

In this study, we want to understand whether using a PLR affects the pain with deep penetration that people with endometriosis experience. To do this, we are asking you to answer the questions in this diary every day for the next 10 weeks.

For the first 4 weeks (i.e. week 1 to week 4), you and your partner can have sex like you normally do. Please use the daily diary circle answer these questions on the following pages:

- Did you have sex? (yes/no)
- How painful was deep penetration? (0 - no pain, to 10

- worst pain imaginable, or N/A if you did not have sex that day)

For the next 6 weeks (week 5 to week 10), you and your partner can have sex with the PLR, which the research team will send to you by mail. Using this daily diary, please circle answer these questions on the following pages:

- Did you have sex? (yes/no)
- Did you use the PLR? (yes/no)
- Did you use lube with the PLR? (yes/no)
- Who put the PLR on your partner? (me/them/together)
- How many rings did you use? (1-4)
- How painful was deep penetration? (0 - no pain, to 10

- worst pain imaginable)

- Did you have any undesirable experiences using the PLR? (yes/no)

Page **18** of **36**

- When we ask if you had sex, we are asking if you had specifically penetrative, vaginal sex (was something like a penis, fingers, or sex toy inserted inside your vagina).
- Fill out the diary every day, even if you and your partner do not have sex.
- On days when you and your partner do not have sex, only answer the question “Did you have sex?”
- On days when you and your partner do not use the PLR, only answer questions “Did you have sex?” and “How painful was deep penetration?”
- If you make a mistake and need more pages, there are extras at the very end of this diary.

Page **19** of **36**

**Daily Diary: Weeks 1-4**

| **Week 1**  **DAY 1 DATE:**  **Please do not use the PLR until WEEK 5, even if it was already delivered by mail.** | | | | | | | | | | | | | |
| --- | --- | --- | --- | --- | --- | --- | --- | --- | --- | --- | --- | --- | --- |
|  | **Did you have sex?** | | **How painful was deep penetration?** | | | | | | | | | | |
| **Day 1** | **Yes** | **No** | **0**  (no pain) | **1** | **2** | **3** | **4** | **5** | **6** | **7** | **8** | **9** | **10**  (worst pain imaginable) |
| **Day 2** | **Yes** | **No** | **0** | **1** | **2** | **3** | **4** | **5** | **6** | **7** | **8** | **9** | **10** |
| **Day 3** | **Yes** | **No** | **0** | **1** | **2** | **3** | **4** | **5** | **6** | **7** | **8** | **9** | **10** |
| **Day 4** | **Yes** | **No** | **0** | **1** | **2** | **3** | **4** | **5** | **6** | **7** | **8** | **9** | **10** |
| **Day 5** | **Yes** | **No** | **0** | **1** | **2** | **3** | **4** | **5** | **6** | **7** | **8** | **9** | **10** |
| **Day 6** | **Yes** | **No** | **0** | **1** | **2** | **3** | **4** | **5** | **6** | **7** | **8** | **9** | **10** |
| **Day 7** | **Yes** | **No** | **0** | **1** | **2** | **3** | **4** | **5** | **6** | **7** | **8** | **9** | **10** |
| **Thank you for completing this week! Please take a photo of this page and email a copy to the research team.** | | | | | | | | | | | | | |

| **Week 2**  **DAY 1 DATE:**  **Please do not use the PLR until WEEK 5, even if it was already delivered by mail.** | | | | | | | | | | | | | |
| --- | --- | --- | --- | --- | --- | --- | --- | --- | --- | --- | --- | --- | --- |
|  | **Did you have sex?** | | **How painful was deep penetration?** | | | | | | | | | | |
| **Day 1** | **Yes** | **No** | **0**  (no pain) | **1** | **2** | **3** | **4** | **5** | **6** | **7** | **8** | **9** | **10**  (worst pain imaginable) |
| **Day 2** | **Yes** | **No** | **0** | **1** | **2** | **3** | **4** | **5** | **6** | **7** | **8** | **9** | **10** |
| **Day 3** | **Yes** | **No** | **0** | **1** | **2** | **3** | **4** | **5** | **6** | **7** | **8** | **9** | **10** |
| **Day 4** | **Yes** | **No** | **0** | **1** | **2** | **3** | **4** | **5** | **6** | **7** | **8** | **9** | **10** |
| **Day 5** | **Yes** | **No** | **0** | **1** | **2** | **3** | **4** | **5** | **6** | **7** | **8** | **9** | **10** |
| **Day 6** | **Yes** | **No** | **0** | **1** | **2** | **3** | **4** | **5** | **6** | **7** | **8** | **9** | **10** |
| **Day 7** | **Yes** | **No** | **0** | **1** | **2** | **3** | **4** | **5** | **6** | **7** | **8** | **9** | **10** |
| **Thank you for completing this week! Please take a photo of this page and email a copy to the research team.** | | | | | | | | | | | | | |

| **Week 3**  **DAY 1 DATE:**  **Please do not use the PLR until WEEK 5, even if it was already delivered by mail.** | | | | | | | | | | | | | |
| --- | --- | --- | --- | --- | --- | --- | --- | --- | --- | --- | --- | --- | --- |
|  | **Did you have sex?** | | **How painful was deep penetration?** | | | | | | | | | | |
| **Day 1** | **Yes** | **No** | **0**  (no pain) | **1** | **2** | **3** | **4** | **5** | **6** | **7** | **8** | **9** | **10**  (worst pain imaginable) |
| **Day 2** | **Yes** | **No** | **0** | **1** | **2** | **3** | **4** | **5** | **6** | **7** | **8** | **9** | **10** |
| **Day 3** | **Yes** | **No** | **0** | **1** | **2** | **3** | **4** | **5** | **6** | **7** | **8** | **9** | **10** |
| **Day 4** | **Yes** | **No** | **0** | **1** | **2** | **3** | **4** | **5** | **6** | **7** | **8** | **9** | **10** |
| **Day 5** | **Yes** | **No** | **0** | **1** | **2** | **3** | **4** | **5** | **6** | **7** | **8** | **9** | **10** |
| **Day 6** | **Yes** | **No** | **0** | **1** | **2** | **3** | **4** | **5** | **6** | **7** | **8** | **9** | **10** |
| **Day 7** | **Yes** | **No** | **0** | **1** | **2** | **3** | **4** | **5** | **6** | **7** | **8** | **9** | **10** |
| **Thank you for completing this week! Please take a photo of this page and email a copy to the research team.** | | | | | | | | | | | | | |

| **Week 4**  **DAY 1 DATE:**  **Please do not use the PLR until WEEK 5, even if it was already delivered by mail.** | | | | | | | | | | | | | |
| --- | --- | --- | --- | --- | --- | --- | --- | --- | --- | --- | --- | --- | --- |
|  | **Did you have sex?** | | **How painful was deep penetration?** | | | | | | | | | | |
| **Day 1** | **Yes** | **No** | **0**  (no pain) | **1** | **2** | **3** | **4** | **5** | **6** | **7** | **8** | **9** | **10**  (worst pain imaginable) |
| **Day 2** | **Yes** | **No** | **0** | **1** | **2** | **3** | **4** | **5** | **6** | **7** | **8** | **9** | **10** |
| **Day 3** | **Yes** | **No** | **0** | **1** | **2** | **3** | **4** | **5** | **6** | **7** | **8** | **9** | **10** |
| **Day 4** | **Yes** | **No** | **0** | **1** | **2** | **3** | **4** | **5** | **6** | **7** | **8** | **9** | **10** |
| **Day 5** | **Yes** | **No** | **0** | **1** | **2** | **3** | **4** | **5** | **6** | **7** | **8** | **9** | **10** |
| **Day 6** | **Yes** | **No** | **0** | **1** | **2** | **3** | **4** | **5** | **6** | **7** | **8** | **9** | **10** |
| **Day 7** | **Yes** | **No** | **0** | **1** | **2** | **3** | **4** | **5** | **6** | **7** | **8** | **9** | **10** |
| **Thank you for completing this week! Please take a photo of this page and email a copy to the research team.** | | | | | | | | | | | | | |

**PLR Instructions**

You have been provided a phallus length reducer (PLR; brand name OhNut) that includes four stackable rings. The rings fit on the base of the penis or penetrating object to limit the depth of vaginal penetration. The rings can be placed on the penis or penetrating object by you, your partner or together.

Please follow the instructions below for putting on the PLR:

1. If you are using a condom, roll the condom onto the penis **before** putting the PLR on.
2. Stretch the PLR - look how far it stretches!


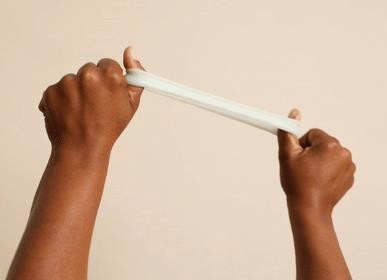


1. Always use lubricant with the PLR. You can use the lubricant you received with the PLR on the entire penis or penetrating object. If you want, you can also use your own silicone or water-based lubricant (but no oil please!). If using a condom, the lubricant needs to go **over** the condom (not under it).

Page **24** of **36**

1. The penis needs to be erect or semi-erect to put the rings on. It is normal to lose some rigidity or hardness temporarily when putting the rings on. But don’t worry, the rigidity can come back during your usual sexual activities!
2. Slide one ring down to the base of the penis or penetrating object at a time. Use as many as you feel comfortable with to start (recommend starting with 3).


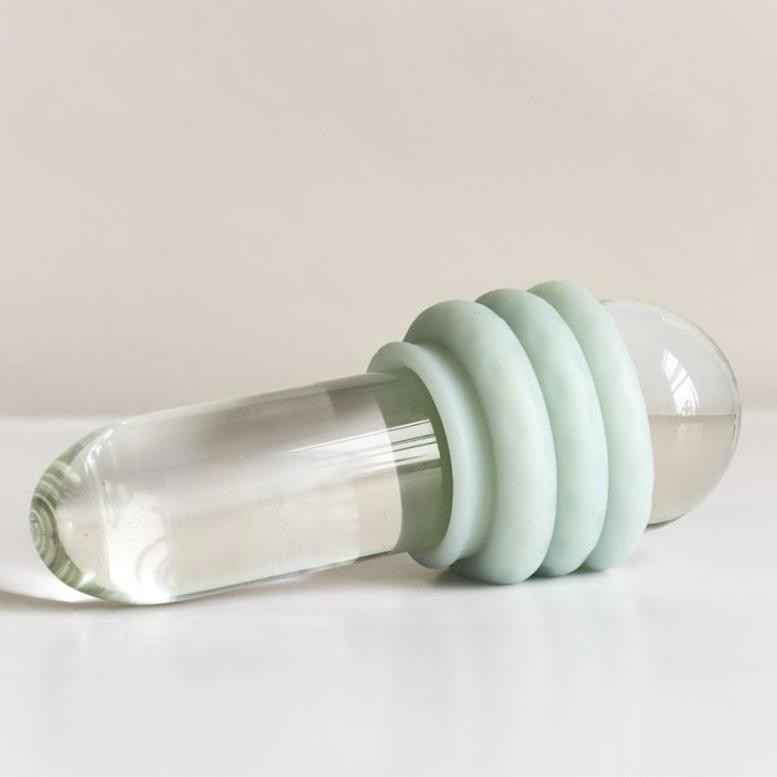


You are ready to go! Remember:

- The PLR is a buffer that limits depth of penetration. This means that the PLR does NOT go inside of the vagina, it should always be outside of your body.
- You can add or remove rings to adjust the depth of penetration based on what you and your partner feel is most comfortable.
- Record how many rings you used and answer the other questions in the daily diary.

Page **25** of **36**

**Daily Diary: Weeks 5-10**

| **Week 5**  **DAY 1 DATE:**   - **Please finish the questionnaires that the research team emailed you before you start using the PLR.** - **Please try to use the PLR as often as possible when you and your partner have sex.** - **You should always use lube with the PLR.** | | | | | | | | | | | | | | | | | | | | | | | |
| --- | --- | --- | --- | --- | --- | --- | --- | --- | --- | --- | --- | --- | --- | --- | --- | --- | --- | --- | --- | --- | --- | --- | --- |
|  | **Did you have sex?** | | **Did you use the PLR?** | | **Did you use lube?** | | **Who put the PLR on your partner?** | **How many rings did you use?** | | | | **How painful was deep penetration?** | | | | | | | | | | | **Did you or your partner have any undesirable experiences when using the PLR?**  ***E.g. Loss of erection, pain, irritation*** |
| Day 1 | **Y** | **N** | **Y** | **N** | **Y** | **N** |  | **1** | **2** | **3** | **4** | **0 1 2**  (no pain) | | | **3** | **4** | **5** | **6** | **7 8 9 10**  (worst pain imaginable) | | | |  |
| Day 2 | **Y** | **N** | **Y** | **N** | **Y** | **N** |  | **1** | **2** | **3** | **4** | **0** | **1** | **2** | **3** | **4** | **5** | **6** | **7** | **8** | **9** | **10** |  |
| Day 3 | **Y** | **N** | **Y** | **N** | **Y** | **N** |  | **1** | **2** | **3** | **4** | **0** | **1** | **2** | **3** | **4** | **5** | **6** | **7** | **8** | **9** | **10** |  |
| Day 4 | **Y** | **N** | **Y** | **N** | **Y** | **N** |  | **1** | **2** | **3** | **4** | **0** | **1** | **2** | **3** | **4** | **5** | **6** | **7** | **8** | **9** | **10** |  |
| Day 5 | **Y** | **N** | **Y** | **N** | **Y** | **N** |  | **1** | **2** | **3** | **4** | **0** | **1** | **2** | **3** | **4** | **5** | **6** | **7** | **8** | **9** | **10** |  |
| Day 6 | **Y** | **N** | **Y** | **N** | **Y** | **N** |  | **1** | **2** | **3** | **4** | **0** | **1** | **2** | **3** | **4** | **5** | **6** | **7** | **8** | **9** | **10** |  |
| Day 7 | **Y** | **N** | **Y** | **N** | **Y** | **N** |  | **1** | **2** | **3** | **4** | **0** | **1** | **2** | **3** | **4** | **5** | **6** | **7** | **8** | **9** | **10** |  |
| **Thank you for completing this week! Please take a photo of this page and email a copy to the research team.** | | | | | | | | | | | | | | | | | | | | | | | |

| **Week 6**  **DAY 1 DATE:**   - **Please finish the questionnaires that the research team emailed you before you start using the PLR.** - **Please try to use the PLR as often as possible when you and your partner have sex.** - **You should always use lube with the PLR.** | | | | | | | | | | | | | | | | | | | | | | | |
| --- | --- | --- | --- | --- | --- | --- | --- | --- | --- | --- | --- | --- | --- | --- | --- | --- | --- | --- | --- | --- | --- | --- | --- |
|  | **Did you have sex?** | | **Did you use the PLR?** | | **Did you use lube?** | | **Who put the PLR on your partner?** | **How many rings did you use?** | | | | **How painful was deep penetration?** | | | | | | | | | | | **Did you or your partner have any undesirable experiences when using the PLR?**  ***E.g. Loss of erection, pain, irritation*** |
| Day 1 | **Y** | **N** | **Y** | **N** | **Y** | **N** |  | **1** | **2** | **3** | **4** | **0 1 2**  (no pain) | | | **3** | **4** | **5** | **6** | **7 8 9 10**  (worst pain imaginable) | | | |  |
| Day 2 | **Y** | **N** | **Y** | **N** | **Y** | **N** |  | **1** | **2** | **3** | **4** | **0** | **1** | **2** | **3** | **4** | **5** | **6** | **7** | **8** | **9** | **10** |  |
| Day 3 | **Y** | **N** | **Y** | **N** | **Y** | **N** |  | **1** | **2** | **3** | **4** | **0** | **1** | **2** | **3** | **4** | **5** | **6** | **7** | **8** | **9** | **10** |  |
| Day 4 | **Y** | **N** | **Y** | **N** | **Y** | **N** |  | **1** | **2** | **3** | **4** | **0** | **1** | **2** | **3** | **4** | **5** | **6** | **7** | **8** | **9** | **10** |  |
| Day 5 | **Y** | **N** | **Y** | **N** | **Y** | **N** |  | **1** | **2** | **3** | **4** | **0** | **1** | **2** | **3** | **4** | **5** | **6** | **7** | **8** | **9** | **10** |  |
| Day 6 | **Y** | **N** | **Y** | **N** | **Y** | **N** |  | **1** | **2** | **3** | **4** | **0** | **1** | **2** | **3** | **4** | **5** | **6** | **7** | **8** | **9** | **10** |  |
| Day 7 | **Y** | **N** | **Y** | **N** | **Y** | **N** |  | **1** | **2** | **3** | **4** | **0** | **1** | **2** | **3** | **4** | **5** | **6** | **7** | **8** | **9** | **10** |  |
| **Thank you for completing this week! Please take a photo of this page and email a copy to the research team.** | | | | | | | | | | | | | | | | | | | | | | | |

| **Week 7**  **DAY 1 DATE:**   - **Please finish the questionnaires that the research team emailed you before you start using the PLR.** - **Please try to use the PLR as often as possible when you and your partner have sex.** - **You should always use lube with the PLR.** | | | | | | | | | | | | | | | | | | | | | | | |
| --- | --- | --- | --- | --- | --- | --- | --- | --- | --- | --- | --- | --- | --- | --- | --- | --- | --- | --- | --- | --- | --- | --- | --- |
|  | **Did you have sex?** | | **Did you use the PLR?** | | **Did you use lube?** | | **Who put the PLR on your partner?** | **How many rings did you use?** | | | | **How painful was deep penetration?** | | | | | | | | | | | **Did you or your partner have any undesirable experiences when using the PLR?**  ***E.g. Loss of erection, pain, irritation*** |
| Day 1 | **Y** | **N** | **Y** | **N** | **Y** | **N** |  | **1** | **2** | **3** | **4** | **0 1 2**  (no pain) | | | **3** | **4** | **5** | **6** | **7 8 9 10**  (worst pain imaginable) | | | |  |
| Day 2 | **Y** | **N** | **Y** | **N** | **Y** | **N** |  | **1** | **2** | **3** | **4** | **0** | **1** | **2** | **3** | **4** | **5** | **6** | **7** | **8** | **9** | **10** |  |
| Day 3 | **Y** | **N** | **Y** | **N** | **Y** | **N** |  | **1** | **2** | **3** | **4** | **0** | **1** | **2** | **3** | **4** | **5** | **6** | **7** | **8** | **9** | **10** |  |
| Day 4 | **Y** | **N** | **Y** | **N** | **Y** | **N** |  | **1** | **2** | **3** | **4** | **0** | **1** | **2** | **3** | **4** | **5** | **6** | **7** | **8** | **9** | **10** |  |
| Day 5 | **Y** | **N** | **Y** | **N** | **Y** | **N** |  | **1** | **2** | **3** | **4** | **0** | **1** | **2** | **3** | **4** | **5** | **6** | **7** | **8** | **9** | **10** |  |
| Day 6 | **Y** | **N** | **Y** | **N** | **Y** | **N** |  | **1** | **2** | **3** | **4** | **0** | **1** | **2** | **3** | **4** | **5** | **6** | **7** | **8** | **9** | **10** |  |
| Day 7 | **Y** | **N** | **Y** | **N** | **Y** | **N** |  | **1** | **2** | **3** | **4** | **0** | **1** | **2** | **3** | **4** | **5** | **6** | **7** | **8** | **9** | **10** |  |
| **Thank you for completing this week! Please take a photo of this page and email a copy to the research team.** | | | | | | | | | | | | | | | | | | | | | | | |

| **Week 8**  **DAY 1 DATE:**   - **Please finish the questionnaires that the research team emailed you before you start using the PLR.** - **Please try to use the PLR as often as possible when you and your partner have sex.** - **You should always use lube with the PLR.** | | | | | | | | | | | | | | | | | | | | | | | |
| --- | --- | --- | --- | --- | --- | --- | --- | --- | --- | --- | --- | --- | --- | --- | --- | --- | --- | --- | --- | --- | --- | --- | --- |
|  | **Did you have sex?** | | **Did you use the PLR?** | | **Did you use lube?** | | **Who put the PLR on your partner?** | **How many rings did you use?** | | | | **How painful was deep penetration?** | | | | | | | | | | | **Did you or your partner have any undesirable experiences when using the PLR?**  ***E.g. Loss of erection, pain, irritation*** |
| Day 1 | **Y** | **N** | **Y** | **N** | **Y** | **N** |  | **1** | **2** | **3** | **4** | **0 1 2**  (no pain) | | | **3** | **4** | **5** | **6** | **7 8 9 10**  (worst pain imaginable) | | | |  |
| Day 2 | **Y** | **N** | **Y** | **N** | **Y** | **N** |  | **1** | **2** | **3** | **4** | **0** | **1** | **2** | **3** | **4** | **5** | **6** | **7** | **8** | **9** | **10** |  |
| Day 3 | **Y** | **N** | **Y** | **N** | **Y** | **N** |  | **1** | **2** | **3** | **4** | **0** | **1** | **2** | **3** | **4** | **5** | **6** | **7** | **8** | **9** | **10** |  |
| Day 4 | **Y** | **N** | **Y** | **N** | **Y** | **N** |  | **1** | **2** | **3** | **4** | **0** | **1** | **2** | **3** | **4** | **5** | **6** | **7** | **8** | **9** | **10** |  |
| Day 5 | **Y** | **N** | **Y** | **N** | **Y** | **N** |  | **1** | **2** | **3** | **4** | **0** | **1** | **2** | **3** | **4** | **5** | **6** | **7** | **8** | **9** | **10** |  |
| Day 6 | **Y** | **N** | **Y** | **N** | **Y** | **N** |  | **1** | **2** | **3** | **4** | **0** | **1** | **2** | **3** | **4** | **5** | **6** | **7** | **8** | **9** | **10** |  |
| Day 7 | **Y** | **N** | **Y** | **N** | **Y** | **N** |  | **1** | **2** | **3** | **4** | **0** | **1** | **2** | **3** | **4** | **5** | **6** | **7** | **8** | **9** | **10** |  |
| **Thank you for completing this week! Please take a photo of this page and email a copy to the research team.** | | | | | | | | | | | | | | | | | | | | | | | |

| **Week 9**  **DAY 1 DATE:**   - **Please finish the questionnaires that the research team emailed you before you start using the PLR.** - **Please try to use the PLR as often as possible when you and your partner have sex.** - **You should always use lube with the PLR.** | | | | | | | | | | | | | | | | | | | | | | | |
| --- | --- | --- | --- | --- | --- | --- | --- | --- | --- | --- | --- | --- | --- | --- | --- | --- | --- | --- | --- | --- | --- | --- | --- |
|  | **Did you have sex?** | | **Did you use the PLR?** | | **Did you use lube?** | | **Who put the PLR on your partner?** | **How many rings did you use?** | | | | **How painful was deep penetration?** | | | | | | | | | | | **Did you or your partner have any undesirable experiences when using the PLR?**  ***E.g. Loss of erection, pain, irritation*** |
| Day 1 | **Y** | **N** | **Y** | **N** | **Y** | **N** |  | **1** | **2** | **3** | **4** | **0 1 2**  (no pain) | | | **3** | **4** | **5** | **6** | **7 8 9 10**  (worst pain imaginable) | | | |  |
| Day 2 | **Y** | **N** | **Y** | **N** | **Y** | **N** |  | **1** | **2** | **3** | **4** | **0** | **1** | **2** | **3** | **4** | **5** | **6** | **7** | **8** | **9** | **10** |  |
| Day 3 | **Y** | **N** | **Y** | **N** | **Y** | **N** |  | **1** | **2** | **3** | **4** | **0** | **1** | **2** | **3** | **4** | **5** | **6** | **7** | **8** | **9** | **10** |  |
| Day 4 | **Y** | **N** | **Y** | **N** | **Y** | **N** |  | **1** | **2** | **3** | **4** | **0** | **1** | **2** | **3** | **4** | **5** | **6** | **7** | **8** | **9** | **10** |  |
| Day 5 | **Y** | **N** | **Y** | **N** | **Y** | **N** |  | **1** | **2** | **3** | **4** | **0** | **1** | **2** | **3** | **4** | **5** | **6** | **7** | **8** | **9** | **10** |  |
| Day 6 | **Y** | **N** | **Y** | **N** | **Y** | **N** |  | **1** | **2** | **3** | **4** | **0** | **1** | **2** | **3** | **4** | **5** | **6** | **7** | **8** | **9** | **10** |  |
| Day 7 | **Y** | **N** | **Y** | **N** | **Y** | **N** |  | **1** | **2** | **3** | **4** | **0** | **1** | **2** | **3** | **4** | **5** | **6** | **7** | **8** | **9** | **10** |  |
| **Thank you for completing this week! Please take a photo of this page and email a copy to the research team.** | | | | | | | | | | | | | | | | | | | | | | | |

| **Week 10**  **DAY 1 DATE:**   - **Please finish the questionnaires that the research team emailed you before you start using the PLR.** - **Please try to use the PLR as often as possible when you and your partner have sex.** - **You should always use lube with the PLR.** | | | | | | | | | | | | | | | | | | | | | | | |
| --- | --- | --- | --- | --- | --- | --- | --- | --- | --- | --- | --- | --- | --- | --- | --- | --- | --- | --- | --- | --- | --- | --- | --- |
|  | **Did you have sex?** | | **Did you use the PLR?** | | **Did you use lube?** | | **Who put the PLR on your partner?** | **How many rings did you use?** | | | | **How painful was deep penetration?** | | | | | | | | | | | **Did you or your partner have any undesirable experiences when using the PLR?**  ***E.g. Loss of erection, pain, irritation*** |
| Day 1 | **Y** | **N** | **Y** | **N** | **Y** | **N** |  | **1** | **2** | **3** | **4** | **0 1 2**  (no pain) | | | **3** | **4** | **5** | **6** | **7 8 9 10**  (worst pain imaginable) | | | |  |
| Day 2 | **Y** | **N** | **Y** | **N** | **Y** | **N** |  | **1** | **2** | **3** | **4** | **0** | **1** | **2** | **3** | **4** | **5** | **6** | **7** | **8** | **9** | **10** |  |
| Day 3 | **Y** | **N** | **Y** | **N** | **Y** | **N** |  | **1** | **2** | **3** | **4** | **0** | **1** | **2** | **3** | **4** | **5** | **6** | **7** | **8** | **9** | **10** |  |
| Day 4 | **Y** | **N** | **Y** | **N** | **Y** | **N** |  | **1** | **2** | **3** | **4** | **0** | **1** | **2** | **3** | **4** | **5** | **6** | **7** | **8** | **9** | **10** |  |
| Day 5 | **Y** | **N** | **Y** | **N** | **Y** | **N** |  | **1** | **2** | **3** | **4** | **0** | **1** | **2** | **3** | **4** | **5** | **6** | **7** | **8** | **9** | **10** |  |
| Day 6 | **Y** | **N** | **Y** | **N** | **Y** | **N** |  | **1** | **2** | **3** | **4** | **0** | **1** | **2** | **3** | **4** | **5** | **6** | **7** | **8** | **9** | **10** |  |
| Day 7 | **Y** | **N** | **Y** | **N** | **Y** | **N** |  | **1** | **2** | **3** | **4** | **0** | **1** | **2** | **3** | **4** | **5** | **6** | **7** | **8** | **9** | **10** |  |
| **Thank you for completing this week! Please take a photo of this page and email a copy to the research team.** | | | | | | | | | | | | | | | | | | | | | | | |

**Thank you**

You have made it to the end of the study! We know that sharing information about your sex life and trying tools to manage pain with deep penetration can feel personal - we are very grateful that you agreed to do this research. We hope that the results of this project will provide knowledge that helps make dealing with pain easier for people with endometriosis in the future.

Before you finish up, please double check to make sure you have:

- Sent copies of all of your pain measurements (page 10 - 16) and daily diaries (page 20 - 23; 26 - 31) to the research team
- Finished all the questionnaires that the research team emailed you

Thank you from the whole research team!

Page **32** of **36**

**Extra Tracking Sheets**

| **Extra sheet for weeks 1 to 4**  **DAY 1 DATE:**  **Please do not use the PLR until WEEK 5, even if it was already delivered by mail.** | | | | | | | | | | | | | |
| --- | --- | --- | --- | --- | --- | --- | --- | --- | --- | --- | --- | --- | --- |
|  | **Did you have sex?** | | **How painful was deep penetration?** | | | | | | | | | | |
| **Day 1** | **Yes** | **No** | **0**  (no pain) | **1** | **2** | **3** | **4** | **5** | **6** | **7** | **8** | **9 10**  (worst pain imaginable) | |
| **Day 2** | **Yes** | **No** | **0** | **1** | **2** | **3** | **4** | **5** | **6** | **7** | **8** | **9** | **10** |
| **Day 3** | **Yes** | **No** | **0** | **1** | **2** | **3** | **4** | **5** | **6** | **7** | **8** | **9** | **10** |
| **Day 4** | **Yes** | **No** | **0** | **1** | **2** | **3** | **4** | **5** | **6** | **7** | **8** | **9** | **10** |
| **Day 5** | **Yes** | **No** | **0** | **1** | **2** | **3** | **4** | **5** | **6** | **7** | **8** | **9** | **10** |
| **Day 6** | **Yes** | **No** | **0** | **1** | **2** | **3** | **4** | **5** | **6** | **7** | **8** | **9** | **10** |
| **Day 7** | **Yes** | **No** | **0** | **1** | **2** | **3** | **4** | **5** | **6** | **7** | **8** | **9** | **10** |
| **Thank you for completing this week! Please take a photo of this page and email a copy to the research team.** | | | | | | | | | | | | | |

| **Extra sheet for weeks 1 to 4**  **DAY 1 DATE:**  **Please do not use the PLR until WEEK 5, even if it was already delivered by mail.** | | | | | | | | | | | | | |
| --- | --- | --- | --- | --- | --- | --- | --- | --- | --- | --- | --- | --- | --- |
|  | **Did you have sex?** | | **How painful was deep penetration?** | | | | | | | | | | |
| **Day 1** | **Yes** | **No** | **0**  (no pain) | **1** | **2** | **3** | **4** | **5** | **6** | **7** | **8** | **9 10**  (worst pain imaginable) | |
| **Day 2** | **Yes** | **No** | **0** | **1** | **2** | **3** | **4** | **5** | **6** | **7** | **8** | **9** | **10** |
| **Day 3** | **Yes** | **No** | **0** | **1** | **2** | **3** | **4** | **5** | **6** | **7** | **8** | **9** | **10** |
| **Day 4** | **Yes** | **No** | **0** | **1** | **2** | **3** | **4** | **5** | **6** | **7** | **8** | **9** | **10** |
| **Day 5** | **Yes** | **No** | **0** | **1** | **2** | **3** | **4** | **5** | **6** | **7** | **8** | **9** | **10** |
| **Day 6** | **Yes** | **No** | **0** | **1** | **2** | **3** | **4** | **5** | **6** | **7** | **8** | **9** | **10** |
| **Day 7** | **Yes** | **No** | **0** | **1** | **2** | **3** | **4** | **5** | **6** | **7** | **8** | **9** | **10** |
| **Thank you for completing this week! Please take a photo of this page and email a copy to the research team.** | | | | | | | | | | | | | |

| **Extra sheet for weeks 5 to 10**  **DAY 1 DATE:**   - **Please finish the questionnaires that the research team emailed you before you start using the PLR.** - **Please try to use the PLR as often as possible when you and your partner have sex.** - **You should always use lube with the PLR.** | | | | | | | | | | | | | | | | | | | | | | | |
| --- | --- | --- | --- | --- | --- | --- | --- | --- | --- | --- | --- | --- | --- | --- | --- | --- | --- | --- | --- | --- | --- | --- | --- |
|  | **Did you have sex?** | | **Did you use the PLR?** | | **Did you use lube?** | | **Who put the PLR on your partner?** | **How many rings did you use?** | | | | **How painful was deep penetration?** | | | | | | | | | | | **Did you or your partner have any undesirable experiences when using the PLR?**  ***E.g. Loss of erection, pain, irritation*** |
| Day 1 | **Y** | **N** | **Y** | **N** | **Y** | **N** |  | **1** | **2** | **3** | **4** | **0 1 2**  (no pain) | | | **3** | **4** | **5** | **6** | **7** | **8 9 10**  (worst pain imaginable) | | |  |
| Day 2 | **Y** | **N** | **Y** | **N** | **Y** | **N** |  | **1** | **2** | **3** | **4** | **0** | **1** | **2** | **3** | **4** | **5** | **6** | **7** | **8** | **9** | **10** |  |
| Day 3 | **Y** | **N** | **Y** | **N** | **Y** | **N** |  | **1** | **2** | **3** | **4** | **0** | **1** | **2** | **3** | **4** | **5** | **6** | **7** | **8** | **9** | **10** |  |
| Day 4 | **Y** | **N** | **Y** | **N** | **Y** | **N** |  | **1** | **2** | **3** | **4** | **0** | **1** | **2** | **3** | **4** | **5** | **6** | **7** | **8** | **9** | **10** |  |
| Day 5 | **Y** | **N** | **Y** | **N** | **Y** | **N** |  | **1** | **2** | **3** | **4** | **0** | **1** | **2** | **3** | **4** | **5** | **6** | **7** | **8** | **9** | **10** |  |
| Day 6 | **Y** | **N** | **Y** | **N** | **Y** | **N** |  | **1** | **2** | **3** | **4** | **0** | **1** | **2** | **3** | **4** | **5** | **6** | **7** | **8** | **9** | **10** |  |
| Day 7 | **Y** | **N** | **Y** | **N** | **Y** | **N** |  | **1** | **2** | **3** | **4** | **0** | **1** | **2** | **3** | **4** | **5** | **6** | **7** | **8** | **9** | **10** |  |
| **Thank you for completing this week! Please take a photo of this page and email a copy to the research team.** | | | | | | | | | | | | | | | | | | | | | | | |

| **Extra sheet for weeks 5 to 10**  **DAY 1 DATE:**   - **Please finish the questionnaires that the research team emailed you before you start using the PLR.** - **Please try to use the PLR as often as possible when you and your partner have sex.** - **You should always use lube with the PLR.** | | | | | | | | | | | | | | | | | | | | | | | |
| --- | --- | --- | --- | --- | --- | --- | --- | --- | --- | --- | --- | --- | --- | --- | --- | --- | --- | --- | --- | --- | --- | --- | --- |
|  | **Did you have sex?** | | **Did you use the PLR?** | | **Did you use lube?** | | **Who put the PLR on your partner?** | **How many rings did you use?** | | | | **How painful was deep penetration?** | | | | | | | | | | | **Did you or your partner have any undesirable experiences when using the PLR?**  ***E.g. Loss of erection, pain, irritation*** |
| Day 1 | **Y** | **N** | **Y** | **N** | **Y** | **N** |  | **1** | **2** | **3** | **4** | **0 1**  (no pain) | | **2** | **3** | **4** | **5** | **6** | **7** | **8 9 10**  (worst pain imaginable) | | |  |
| Day 2 | **Y** | **N** | **Y** | **N** | **Y** | **N** |  | **1** | **2** | **3** | **4** | **0** | **1** | **2** | **3** | **4** | **5** | **6** | **7** | **8** | **9** | **10** |  |
| Day 3 | **Y** | **N** | **Y** | **N** | **Y** | **N** |  | **1** | **2** | **3** | **4** | **0** | **1** | **2** | **3** | **4** | **5** | **6** | **7** | **8** | **9** | **10** |  |
| Day 4 | **Y** | **N** | **Y** | **N** | **Y** | **N** |  | **1** | **2** | **3** | **4** | **0** | **1** | **2** | **3** | **4** | **5** | **6** | **7** | **8** | **9** | **10** |  |
| Day 5 | **Y** | **N** | **Y** | **N** | **Y** | **N** |  | **1** | **2** | **3** | **4** | **0** | **1** | **2** | **3** | **4** | **5** | **6** | **7** | **8** | **9** | **10** |  |
| Day 6 | **Y** | **N** | **Y** | **N** | **Y** | **N** |  | **1** | **2** | **3** | **4** | **0** | **1** | **2** | **3** | **4** | **5** | **6** | **7** | **8** | **9** | **10** |  |
| Day 7 | **Y** | **N** | **Y** | **N** | **Y** | **N** |  | **1** | **2** | **3** | **4** | **0** | **1** | **2** | **3** | **4** | **5** | **6** | **7** | **8** | **9** | **10** |  |
| **Thank you for completing this week! Please take a photo of this page and email a copy to the research team.** | | | | | | | | | | | | | | | | | | | | | | | |


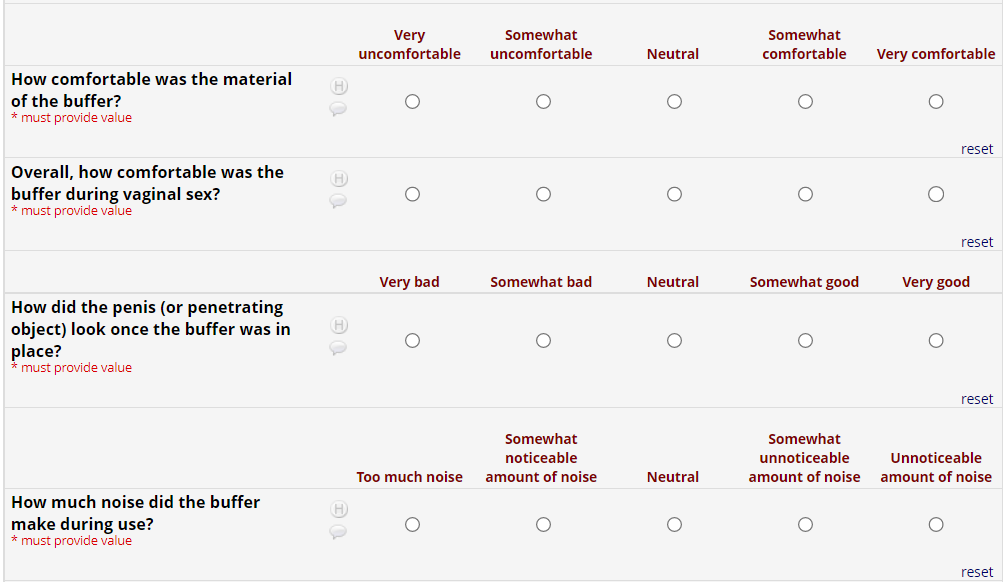

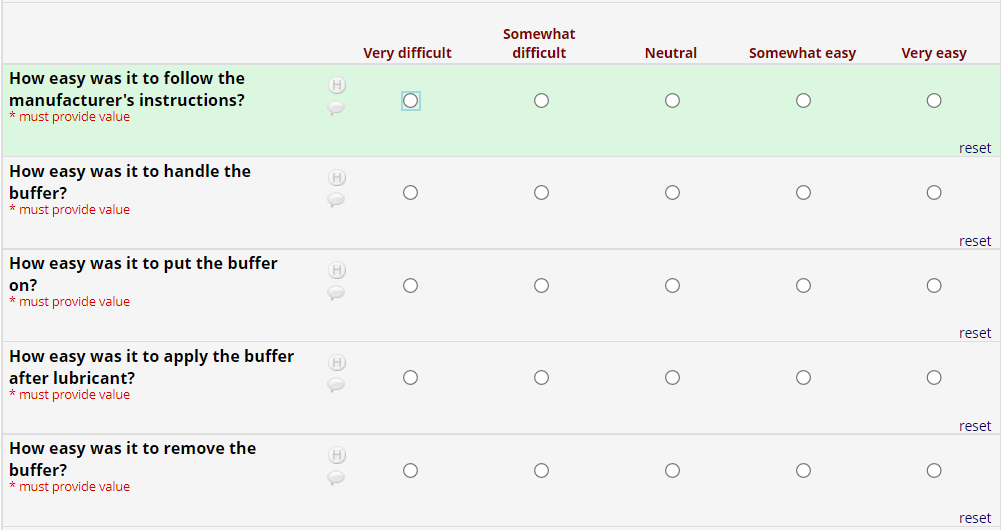
**Appendix B: Patient PLR Feedback Questionnaire**


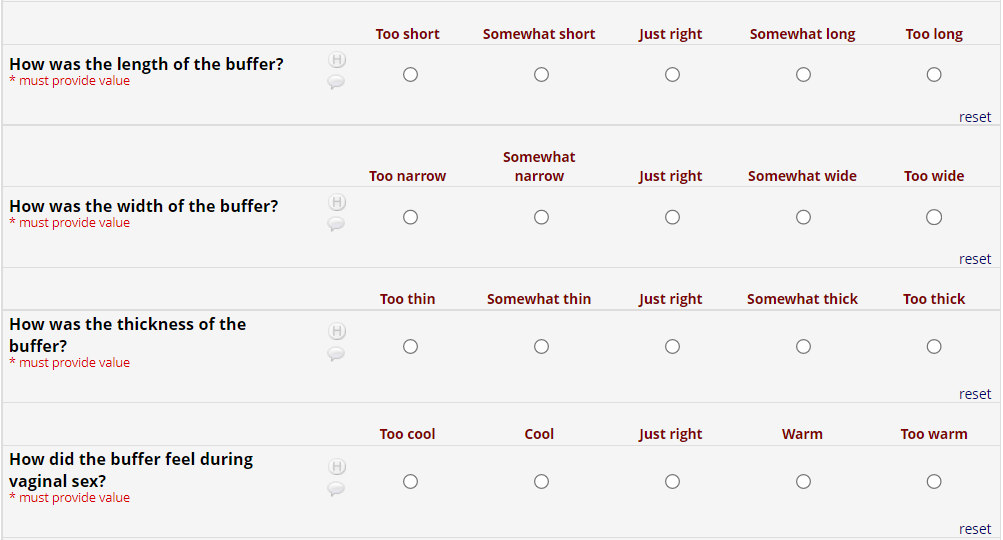

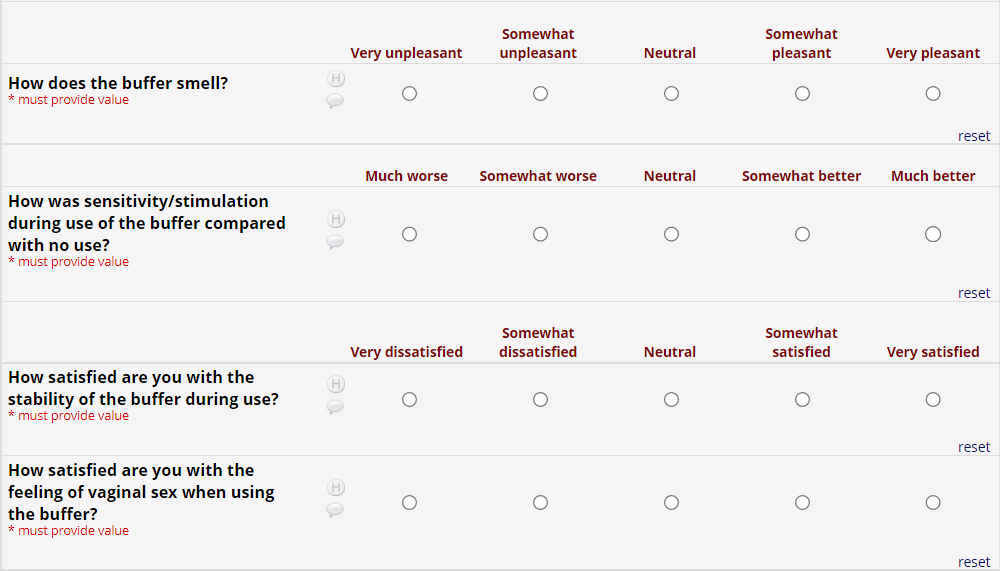


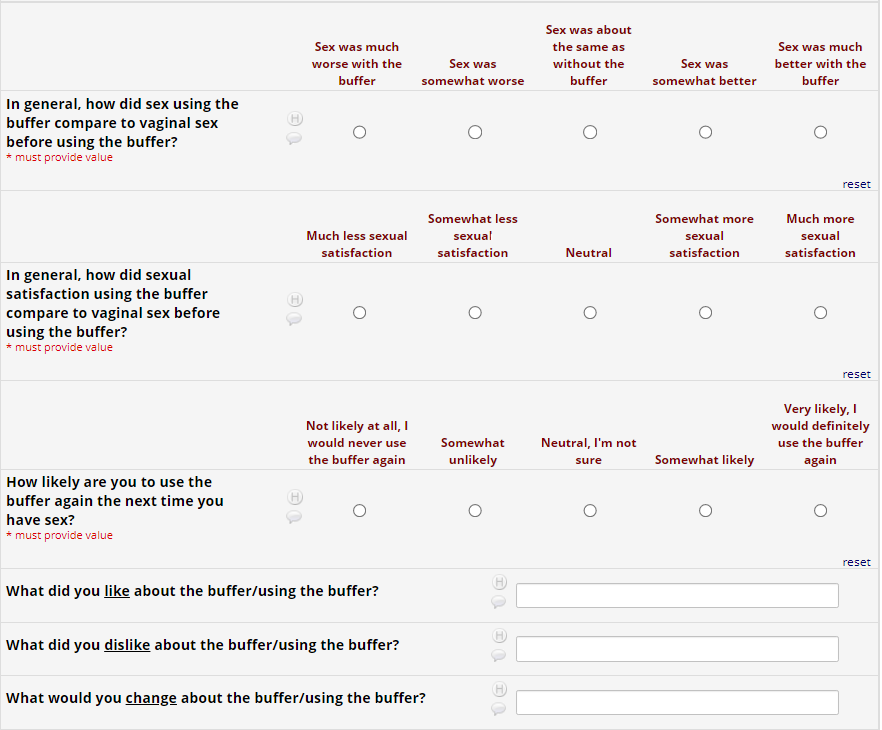


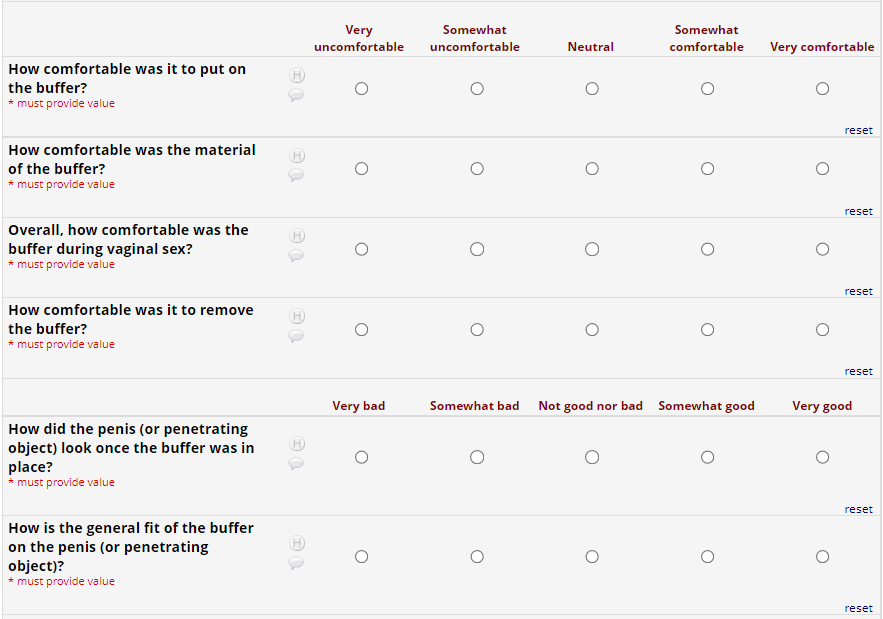

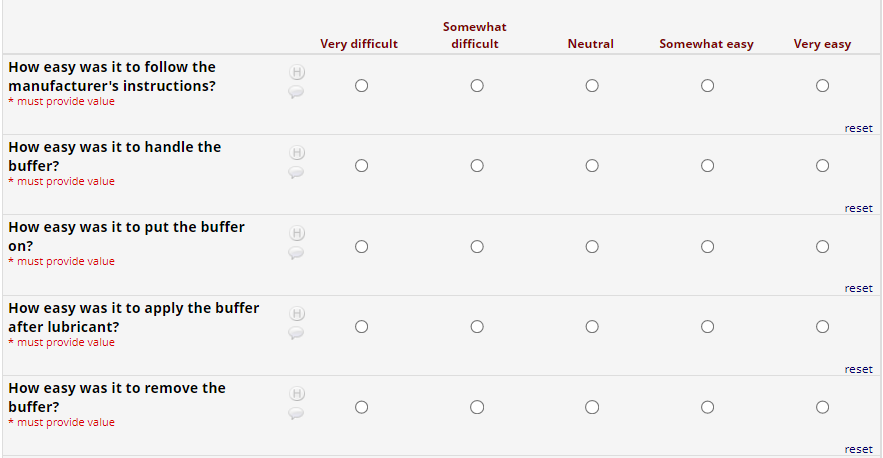
**Appendix B: Partner PLR Feedback Questionnaire**


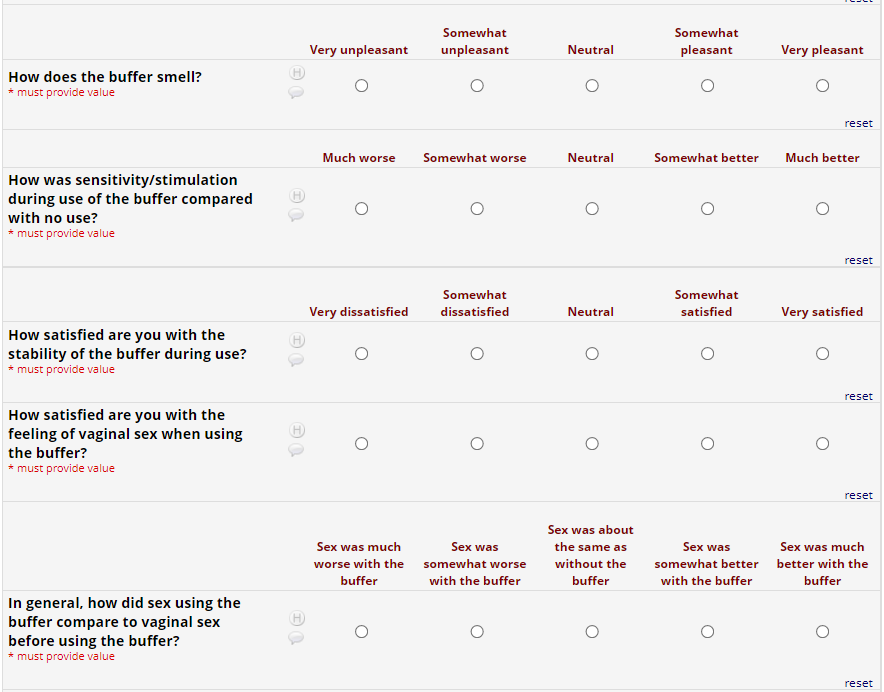

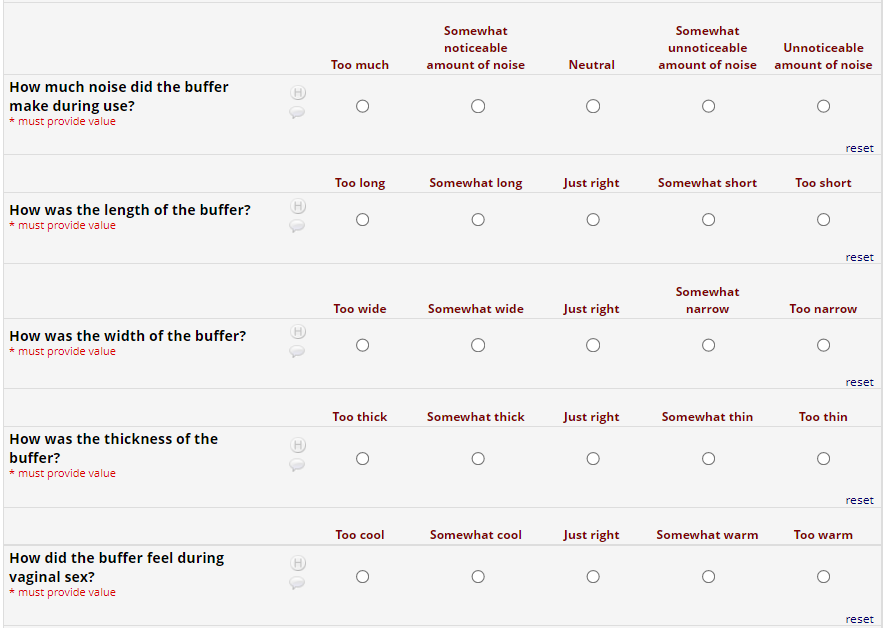


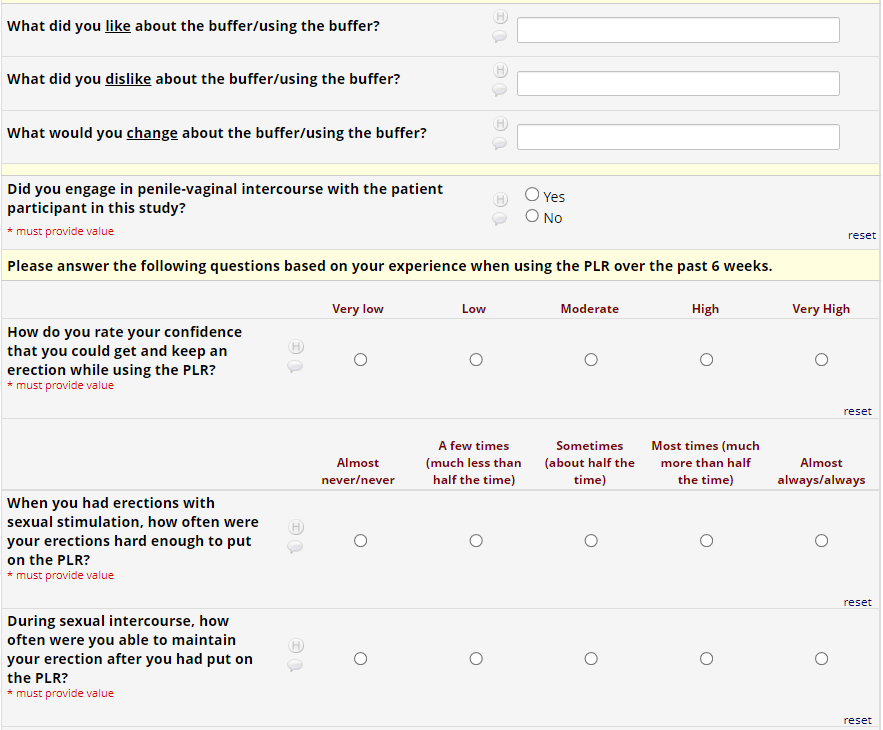

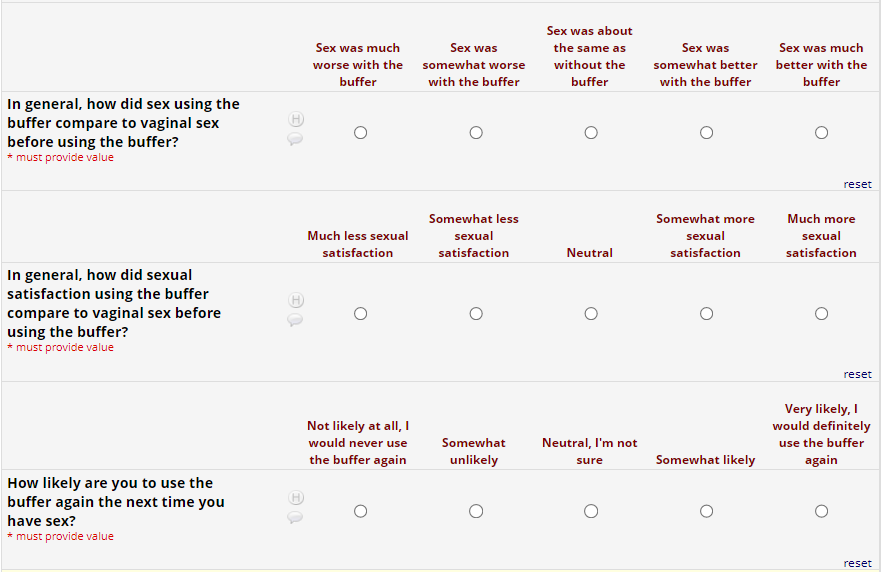


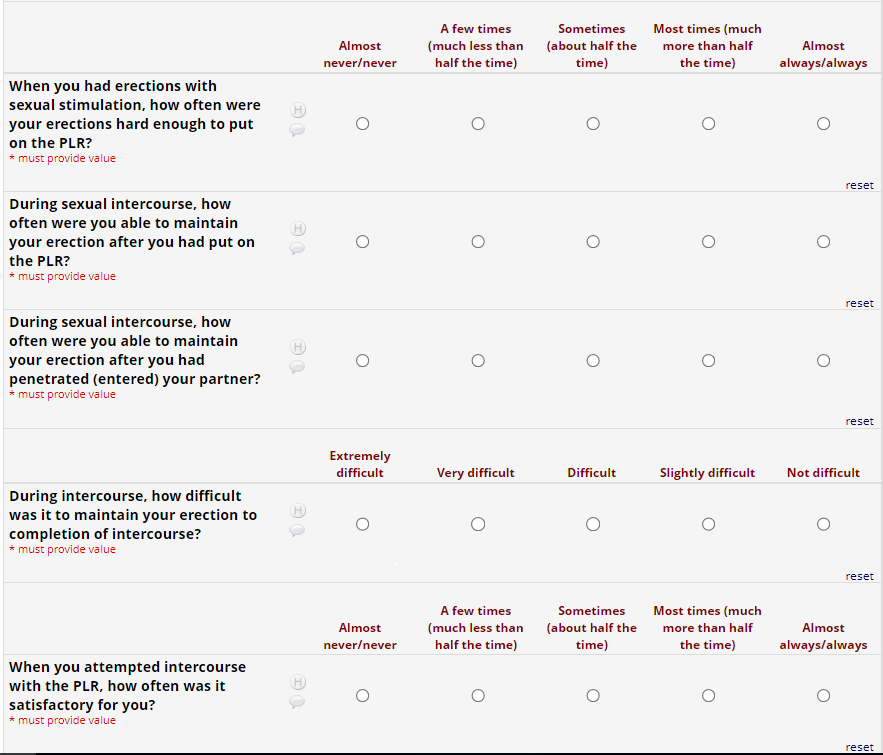


**Appendix C: Patient Vaginal Insert Feedback Questionnaire**


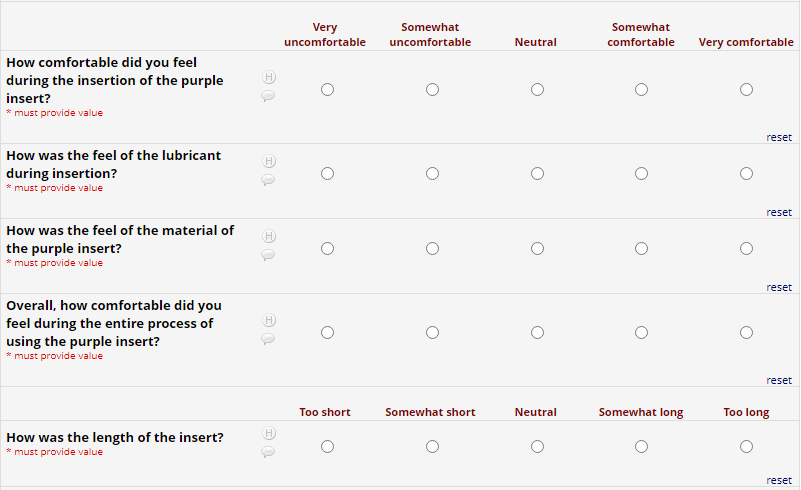

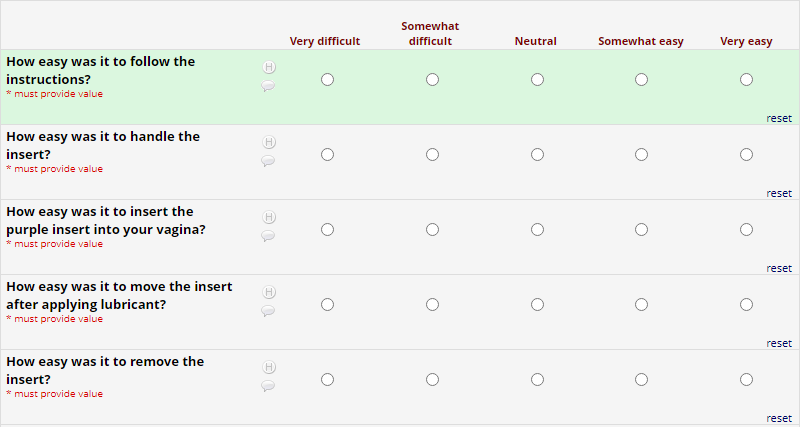


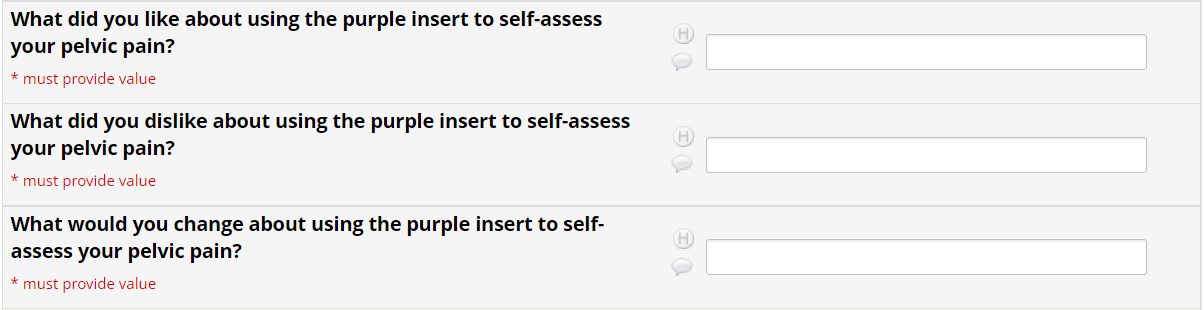

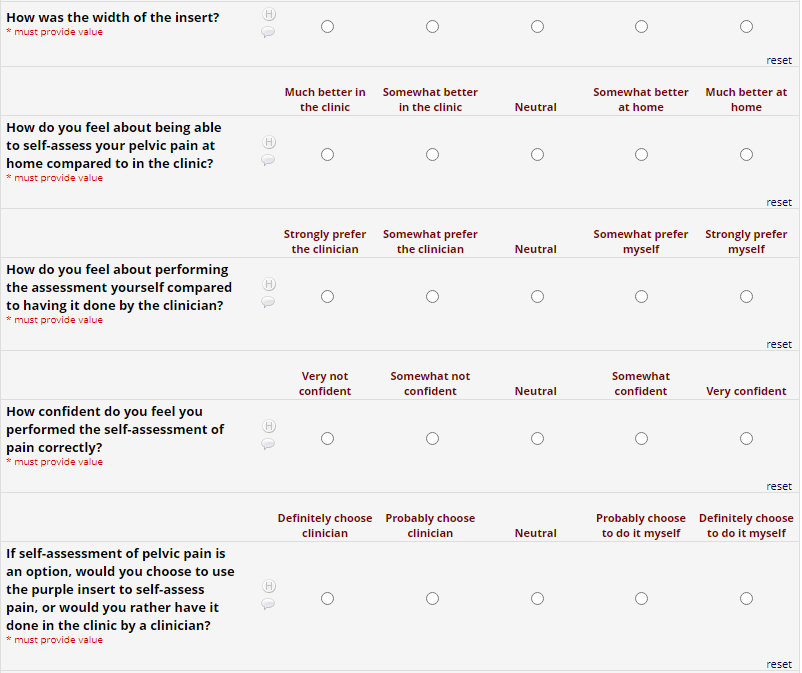

Supplement: Multimedia Appendix 1 [file resprot_v12i1e39834_app1.docx]
